# Methodological Approaches to Comparative Trend Analyses: The Case of Adolescent Toothbrushing

**DOI:** 10.3389/ijph.2024.1607669

**Published:** 2025-01-10

**Authors:** Torbjørn Torsheim, Frank J. Elgar, Alina Cosma, Caroline Residori, Oddrun Samdal, Christina Schnohr

**Affiliations:** ^1^ Department of Psychosocial Science, University of Bergen, Bergen, Norway; ^2^ Department of Equity, Ethics and Policy, School of Population and Global Health, McGill University, Montreal, QC, Canada; ^3^ Trinity Research in Childhood Centre, School of Psychology, Trinity College Dublin, Dublin, Ireland; ^4^ Olomouc University Social Health Institute, Palacky University Olomouc, Olomouc, Czechia; ^5^ Department of Social Sciences, Faculty of Humanities, Education and Social Sciences, University of Luxembourg, Esch-sur-Alzette, Luxembourg; ^6^ Department of Health Promotion and Development, University of Bergen, Bergen, Norway; ^7^ Department of Public Health, University of Copenhagen, Copenhagen, Denmark; ^8^ Centre for Public Health Research in Greenland, National Institute of Public Health, University of Southern Denmark, Odense, Denmark

**Keywords:** HBSC study, trend analysis, methodological research, comparative analyses, toothbrushing

## Abstract

**Objectives:**

Research questions about how and why health trends differ between populations require decisions about data analytic procedure. The objective was to document and compare the information returned from stratified, fixed effect and random effect approaches to data modelling for two prototypical descriptive research questions about comparative trends in toothbrushing.

**Methods:**

Data included five cycles of the Health Behaviour in School-aged Children 2006 to 2022, which provided a sample of 980192 11- to 15- year olds from 35 countries. Using logistic regression models and generalized linear mixed models, toothbrushing daily was regressed on time, following the three approaches to analysis of trends.

**Results:**

The stratified approach suggested a positive but non-linear trend in toothbrushing from 2006 to 2022 in most countries but provided no statistical inference on the variation. The fixed effect and the random effect approach converged on a positive but flattening overall trend, with a statistically significant country variation in trends.

**Conclusion:**

Only the fixed effect approach and the random effects approach provided clear answers to the research question. Additional methodological considerations for making an informed choice of analytical approach are discussed.

## Introduction

In the dynamic landscape of public health, staying abreast of emerging trends in health and health behaviours is paramount for effective policy formulation and implementation. Health trends, characterized by developments in risk behaviours by socio-demographic factors, serve as invaluable indicators of the evolving public health policy, and has received increasing attention as a field of research [1–6].

Adopting a comparative perspective on time trends enables interesting research questions about how and why health trends differ between populations. Such research questions also set strong requirements for study design, measurement, model specification, and choice of data analytic procedure. The “Health Behaviour in School-aged Children study (HBSC)” has a research design that is highly relevant for research questions about time trends in health. In the HBSC study, survey data collection is repeated every 4 years, on independent samples of new cohorts of 11– to 15–years olds from the same countries or regions. The study has a repeated structure at the country/region-level with country A, B, and C measured at several time points, but a cross-sectional structure at the individual-level. This design allows for tests about how societies change, but not how individuals change.

Previous methodological papers have addressed the unique challenges related to obtaining comparability of research protocols, sampling frames, and measurement [7–9], but the required data analytic decisions have received less attention. The current study highlights the choice of analytic approach in empirical analyses of comparative trends.

The generic class of regression models provide a flexible analytical framework for comparative time trend analysis. In such models, a health outcome is the dependent variable for the independent variable historical time. For a simple linear model, the trend can be summarised through a single parameter: the regression coefficient of change per time unit.

Trends are not always linear, and specification of the shape of the trend is a central task in comparative trend analysis. When there are three or more cycles of data, non-linear time trends can be fitted through higher-order polynomials, including quadratic and cubic terms of time. A simple linear shape makes a direct interpretation possible, where the trend can be translated into an “increase” or “decrease” over time. When the model include quadratic and cubic effects, the trend is a composite of effects, and difficult to interpret directly form the regression model coefficients. To interpret non-linear trends, obtained model predictions can provide the necessary information.

A challenge particular to comparative time trend studies is how to model and test *country differences* of trends. By focussing on regression model-based tests of trends, we have identified three major comparative approaches: the stratified approach, the fixed effect approach, and the random effects approach to trends.

The *stratified approach* involves running a series of regression analyses split by country, regressing the relevant health outcome with time as the focal independent variable. A common model is specified and repeated for each country. With a dichotomous health outcome as dependent variable and time as continuous independent variable the generalized linear regression model for binomial data with a logit link becomes:

$$\text{logit}\left(P\right)=\ln\left(\frac{P}{1-P}\right)=\beta_{0}+\beta_{1}time$$

In the *fixed effect approach*, the average trends and country differences of trends can be modelled through specification of main and interaction effects of time and country, where the effect of country is specified through, for example, deviation coding or simple contrast coding. With a simple contrast specification for countries A,B,C this generalized linear model becomes:

$$\text{logit}\left(P\right)=\ln\left(\frac{P}{1-P}\right)=\beta_{0}+\beta_{1}time+\beta_{2}Country\;B+\beta_{3}Country\;C+\beta_{4}Country\;B\times time+\beta_{5}Country\;C\times time$$

where $\beta_{0}$ and $\beta_{1}$ time describe the intercept and effect of time for the reference country A, $\beta_{2}$ and $\beta_{3}$ describe country B and C differences in intercept relative to country A, and $\beta_{4}$ and $\beta_{5}$ are interaction terms describing country B and C difference in the effect of time relative to country A.

In the *random effects approach*, the average trend is modelled as a fixed term ( $\beta_{1}time$ ), but the country differences in such trends are parameterized through random variance components that can be functions of time. The random effects can be structured in several ways [10]. The “societal growth curve specification” is relevant for our purpose [10]. For a comparative repeated cross-sectional study, a three-level generalized linear mixed model can be specified, using person (*i*), country-year (*j*) and country (*k*) as levels within the model:

$$\text{logit}\left(P_{ijk}\right)=\ln\left(\frac{P_{ijk}}{1-P_{ijk}}\right)=\beta_{0_{ijk}}+\beta_{1k}time+u_{0j}+v_{0k}+v_{1k}time$$

This three-level composite specification includes a fixed part intercept $\beta_{0}$ , a fixed effect of time $\beta_{1}$ , and a random part with three components: A random country-year-level (*j*) intercept component $u_{0j}∼N\left(0,\sigma_{u0}^{2}\right)$ capturing fluctuations within country across years; a random country-level (*k*) intercept component $v_{0k}∼N\left(0,\sigma_{v0}^{2}\right)$ capturing country-level differences in the dependent variable, and a country-level random slope component $v_{1k}∼N\left(0,\sigma_{v1}^{2}\right)$ capturing between-country variation in the slope of time.

### The Current Study

With a considerable diversity in types of research questions and available analytical approaches, there is a need to examine the relative utility and relevance of different approaches to comparative time trend analyses in applied research.

In the current study, we demonstrate and compare model information and results from stratified, fixed effect and random effect approaches to comparative trends on a real-data case from the HBSC study: adolescent toothbrushing between 2006 and 2022 in 35 countries. The used data partly overlap with a previous study of trends in toothbrushing [11], but in the current study the primary objective is methodological.

To structure the comparison between approaches, we used each approach to answer two seemingly simple research questions:

Research question 1 (RQ1): Did toothbrushing show an overall linear time trend 2006–2022?
Research question 2 (RQ2): Did countries/regions show different time trends?

## Methods

### Data

The Health Behaviour in School-aged Children study is a large WHO-collaborative school-based survey carried out every 4 years, among a sample of 11-, 13-, and 15-year-olds, with an even distribution of boys and girls. Respondents completed anonymous questionnaires in a class-room setting following a standardized protocol, which has been developed and updated for every survey round. The HBSC protocol is used across all participating countries, ensuring high comparability of data across an increasing number of countries over time and repeated survey rounds. In the current study only data from five of the total 11 cycles of data collection was used, covering the period 2006 to 2022. Open data can be accessed on https://www.uib.no/en/hbscdata/113290/open-access. Countries or regions that took part in all five survey rounds were included, representing a sample of N = 980,192 students from 35 countries or regions, with 50.6% girls, and balanced age category composition. The 35 countries and regions are listed in Table 1.

**TABLE 1:** Sample frequency of toothbrushing twice or more daily in 35 countries and regions in five cycles of the Health Behaviour in School-aged Children study (2006–2022).

Country/region	No	Yes	%
Austria	5,445	17,002	75.7
Belgium (VLG)	9,687	16,279	62.7
Belgium (WAL)	9,706	15,763	61.7
Canada	19,903	39,899	68.4
Croatia	10,052	16,910	62.7
Czech Republic	10,331	28,274	73.3
Denmark	4,872	17,351	78.1
Estonia	7,985	14,193	64.0
Finland	10,468	13,915	57.0
France	8,978	24,151	72.8
Germany	6,034	22,385	78.7
Greece	11,214	11,589	50.8
Greenland	2,352	3,425	59.3
Hungary	8,073	11,838	59.4
Iceland	15,202	32,441	68.1
Ireland	7,008	13,990	66.6
Israel	6,955	13,564	66.9
Italy	5,620	15,886	73.9
Latvia	11,445	12,814	52.8
Lithuania	12,774	12,523	49.5
Luxembourg	5,855	14,223	70.8
Netherlands	4,891	17,219	77.7
Norway	4,566	13,794	75.1
Poland	8,500	16,304	65.7
Portugal	7,498	16,403	68.6
Romania	13,358	13,087	49.5
Slovakia	9,820	15,515	61.2
Slovenia	8,835	18,614	67.5
Spain	11,630	21,395	64.2
Sweden	4,763	22,334	82.4
Switzerland	5,011	27,152	84.4
North Macedonia	8,115	13,947	63.2
England	4,813	16,077	76.7
Scotland	7,111	20,964	74.6
Wales	18,452	47,152	71.8
Total	307,322	648,372	67.9

### Measures

Toothbrushing was measured with a single frequency item: “*How often do you brush your teeth?*” *with* the five response categories (1: “*More than once a day*”; 2: “*Once a day*”; 3: “*At least once a week but not daily*,” 4: “*Less than once a week*” and 5: “*Never*”). In the analyses for the present paper the outcome was defined as “toothbrushing *more than once a day*,” collapsing the four other categories to 0.

### Data Analysis

We used R version 4.4.1 [12] for all statistical analysis and visualization, R stats glm function for the stratified approach and the fixed effect approach, and the lme4 [13] package glmer function for the random effects approach. Model selection was based on Likelihood ratio test (LRT) of nested models and Akaike’s information criterion (AIC) and Bayes information criterion (BIC). Model assumptions for the logistic regression models include no outliers, inclusion of all relevant independent variables, linearity across the prediction, and independence of responses. The logistic regression model with random effects also assumes normal distributed random effects.

We tested logistic regression model assumptions through the random quantile residual function of the STATMOD R package [14]. As compared to Pearsons or Deviance residuals, random quantile residuals are less affected by the scaling of the dependent variable and improves the diagnostic information from analysis of residuals from discrete outcomes [15]. For the random effect approach, we also examined the assumption of normal distributed random effects with normal QQ-plots.

For the stratified approach we used the generalized linear model for binomial data, with a logit link function, also referred to as a logistic regression model. For each region, we regressed the dependent variable toothbrushing on continuous time. Linear (M1), quadratic (M2) and cubic (M3) effects of time was entered blockwise, with one set of analyses per country or region. In all analyses, time was centred at year 2014, to reduce multicollinearity between time, time quadratic, and time cubic. Centred time was rescaled to 10-year unit, making the regression coefficient the change in toothbrushing per 10-year period.

For the *fixed effect* approach, we modelled the average trends and country/region differences through specification of main and interaction effects of time and country/region, using deviation contrasts for country/region. Model M0 included main effects of country/region. Models M1 to M3 included linear, quadratic and cubic effects of time. To test country/region differences in trends (RQ2) we entered country/region by time, country/region by quadratic time, and country/region by cubic time (M4 to M6). Likelihood ratio test of the main effects of time allowed for the assessment of the statistical significance of an overall trend across all countries (RQ1), while the interaction time by country allow for at omnibus test of region differences in trends (RQ2).

The *random effects* approach was implemented through a three-level generalized linear mixed regression model including a constant logistic conditional variance at the student level ( $\frac{\pi^{2}}{3}$ ), and random effects at the region-year and region level. Model M0 was a null model including a fixed intercept ( $\beta_{0}$ ) and random country/region-year $\left[u_{0j}∼N\left(0,\sigma_{u0}^{2}\right)\right]$ and country/region $\left[v_{0k}∼N\left(0,\sigma_{v0}^{2}\right)\right]$ intercept variance components. Models M1 to M3 tested fixed linear ( $\beta_{1}$ ), quadratic ( $\beta_{2}$ ) and cubic time ( $\beta_{3}$ ), relevant to interpret the trend shape, and the overall average trends of toothbrushing (RQ1). Model M4 included a random slope of time at the region level ${\left(V_{1\text{time}}\right)v}_{1k}∼N\left(0,\sigma_{v1}^{2}\right)$ , relevant to our research question about between-region differences in trends in toothbrushing (RQ2). R glmer function uses Laplace approximation when there are multiple levels of random effects. We extracted model-based predictions with the ggeffects package [16]. Assumptions of normal-distributed random effects were examined with diagnostic QQ-plot from sjPlot [17] package.

## Results

Table 1 shows the sample frequency of toothbrushing twice or more often daily per country or region, collapsed over study cycles.

### Stratified Approach

Prior to statistical analysis we inspected the descriptive frequencies of toothbrushing per country and region over time, as shown in Supplementary Figure S1. We note different patterns across countries.

Supplementary Table S1 shows the results of 35 blockwise logistic regression models with toothbrushing as the dependent variable regressed on time, time-quadratic and time-cubic in the stratified approach, with three model blocks (models M1 to M3) per country/region. Supplementary Table S2 shows the model summary statistics Deviance, BIC, AIC and LRT model comparisons for the 35 sets of analyses.

Model diagnostics of quantile residuals for model M3 in the stratified approach revealed no patterns with the linear predictor (Supplementary Figure S2), and the normal QQ plot suggested no residual deviation for any country/region (Supplementary Figure S3).

**FIGURE 1:**
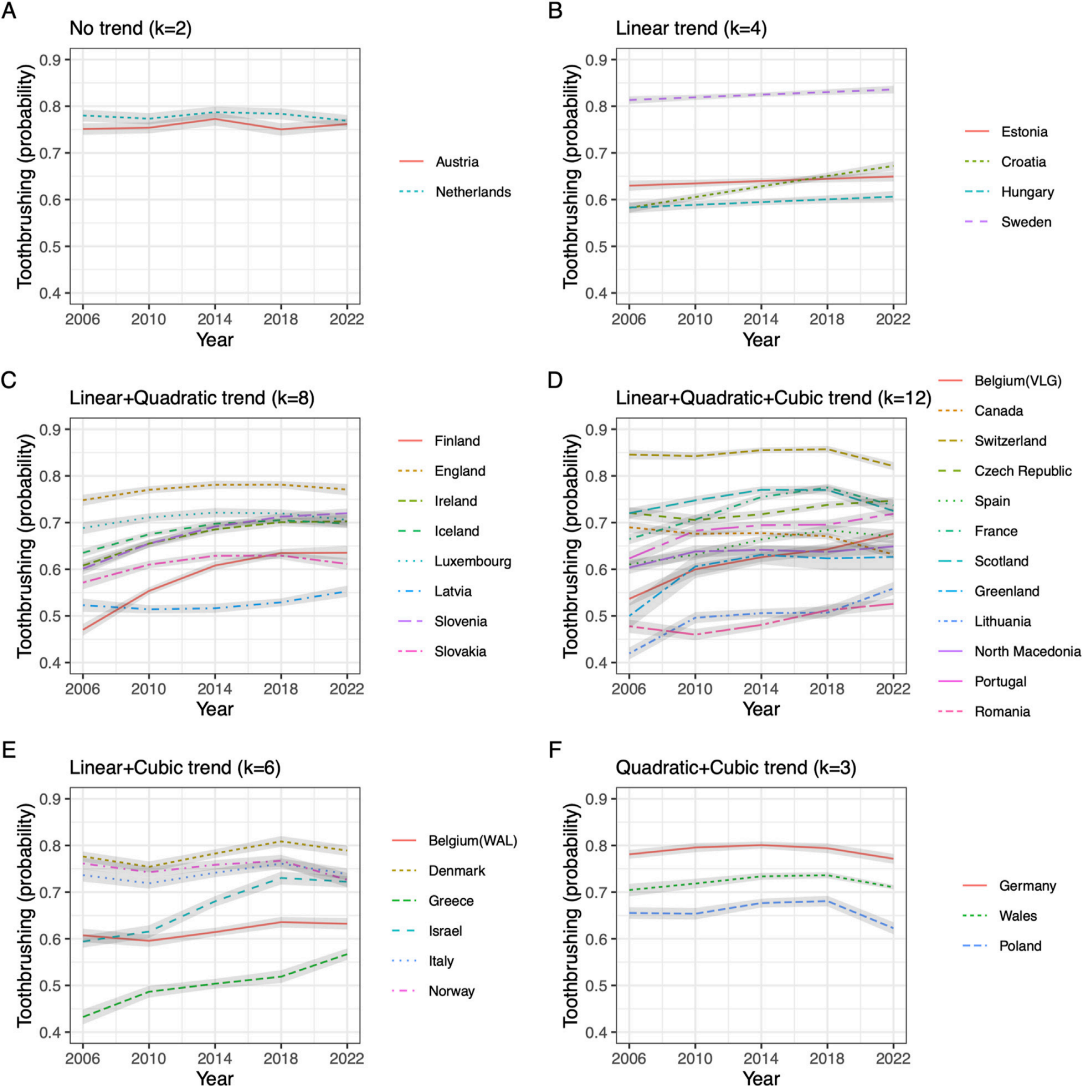
Predicted toothbrushing by time per pattern of trend components, stratified approach. **(A)** Countries with no trend; **(B)** Countries with linear trend; **(C)** Countries with linear and quadratic components; **(D)** Countries with Linear, quadratic and cubic trend components; **(E)** Countries with linear and cubic trend components; **(F)** Countries with quadratic and cubic trend components (Data from Health behaviour in school-aged children 2006–2022).

The LRT difference between models informs about the shape and magnitude of trends, and *post hoc* we used the information to summarize different trend patterns. The profile of trends in the stratified approach is shown in Figures 1A–F. For two countries there were no statistically significant trends (Austria, Netherlands). Four countries (panel B) showed linear trends only (Estonia, Croatia, Hungary, Sweden). For eight countries (Finland, England, Ireland Iceland, Luxembourg, Latvia, Slovenia, and Slovakia) there were statistically significant linear and quadratic blocks (panel C), and for twelve countries blocks of linear, quadratic and cubic components were all statistically significant [panel D: Belgium (VLG), Canada, Switzerland, Czech Republic, Spain, France, Scotland, Greenland, Lithuania, North Macedonia, Portugal, Romania]. Six countries (panel E) showed a pattern of statistically significant linear effects, non-significant quadratic effects, and significant cubic effects [Belgium (WAL), Denmark, Greece, Israel, Italy and Norway]. Three countries (panel F) showed a pattern with significant blocks of quadratic and cubic terms but without a significant linear block (Wales, Poland and Germany).

To summarize, the stratified approach showed a strong diversity of trend shape, with few countries showing a monotone linear trend, but most countries showed a composite positive trend in toothbrushing.

### Fixed Effect Approach

Table 2 shows the model summary for the fixed effect approach. The likelihood ratio test of the difference between nested models revealed statistically significant increments in model fit for linear time (M1), quadratic time (M2) and cubic time (M3), as well as interactions country/region by linear time (M4), country/region by quadratic time (M5), and country/region by cubic time (M6). AIC and the LRT suggested M6 to be the best model, whereas BIC identified model M4 as the best fitting model. The results of model M6 of the fixed effect approach suggested a linear, quadratic and cubic component in the overall trends, and that linear, quadratic and cubic components were different across countries and regions.

**TABLE 2:** Model summary for fixed effects approach. Model comparisons are conducted on likelihood ratio test (LRT) difference test on model difference degrees of freedom, Akaike’s information criterion (AIC) and Bayes information criterion (BIC) (smaller is better). (Health behaviour in school-aged children 2006–2022).

	M0 No trend Country/Region main effects	M1 Linear Country/Region main effects	M2 Quadratic Country/Region main effects	M3 Cubic Country/Region main effects	M4 Linear by Country/Region	M5 Quadratic by Country/Region	M6 Cubic by Country/Region
Parameters	35	36	37	38	72	106	140
AIC	1,167,721.99	1,166,648.29	1,166,370.11	1,166,358.81	1,165,122.03	1,164,902.00	1,164,752.77
BIC	1,168,133.94	1,167,072.01	1,166,805.60	1,166,806.08	1,165,969.48	1,166,149.64	1,166,400.60
Deviance	1,167,651.99	1,166,576.29	1,166,296.11	1,166,282.81	1,164,978.03	1,164,690.00	1,164,472.77
df.residual	955,659	955,658	955,657	955,656	955,622	955,588	955,554
LRT	32,789.16	1,075.70	280.18	13.30	1,304.78	288.03	217.23
df M_k_–M_k.1_	34	1	1	1	34	34	34
*p*-value	<0.001	<0.001	<0.001	<0.001	<0.001	<0.001	<0.001

Note. Null deviance is 1,200,441.1, df = 955,693.

Supplementary Figure S4 include model diagnostics for model M6 fixed effect approach. The quantile residuals for model M6 were constant across the linear prediction (panel A). Residuals did not vary as a function of time (panel B) or country/region (panel C). The quantile-quantile plot (panel D) suggested that there were no outlying cases.

Supplementary Table S3 shows the model coefficients for model M6 for the fixed effect approach. As the trend has three components the single regression coefficients convey limited information about the total trend for a country or region. The model coefficients for time show that at time 0 the mean linear growth rate per decade is 0.19, but the negative quadratic effect of −0.13 and cubic effect of −0.10 indicate that the average growth rate changed across time, levelling off over time. This indicate that the overall trend was non-linear.

The deviation contrasts for the main effect, represent each country/regions difference to the mean level of toothbrushing at time 0 (in our example: 2014), here in logit unit. The B/SE ratio for each deviation contrast is the test statistic for the hypothesis that the specific country/region contrast is different from the mean intercept, or from the mean linear component, the mean quadratic component or the mean cubic component.

**FIGURE 2:**
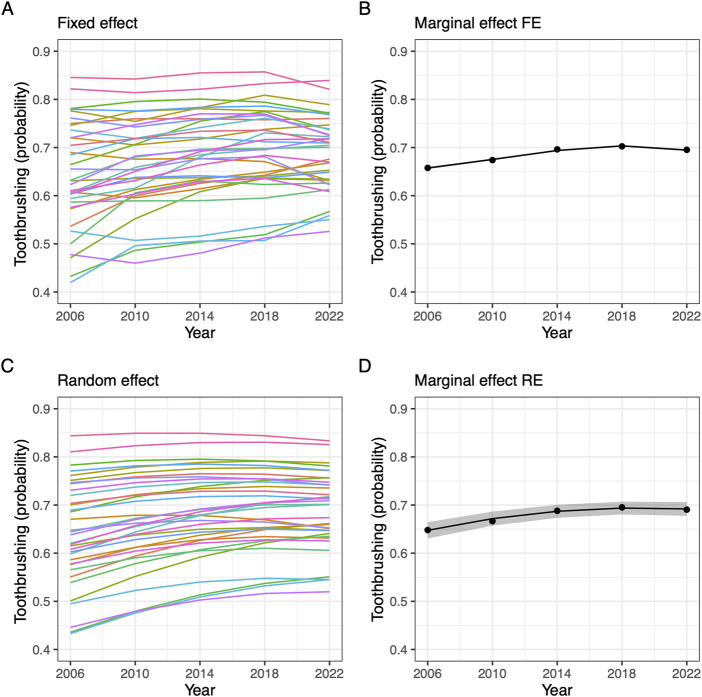
Predicted toothbrushing by time. **(A, B)** Show predictions from fixed effect model, **(C, D)** shows predictions from random effect model. **(A, C)** give predictions per country or region, one line per country/region. **(B, D)** give mean overall prediction, with confidence intervals (Data from Health behaviour in school-aged children 2006–2022).

More specific information about the trends and the differences in trends for specific countries were obtained through model-based predictions and relevant linear composites, and we illustrate these predictions and the random effect predictions in the next section on random effects (Figure 2, panels A, B).

### Random Effects Approach

Table 3 shows the model summaries results from the random effects approach. The likelihood ratio test of nested model differences indicated statistically significant linear (M1) and quadratic (M2) components, but not a cubic component (M3). For model M4, the inclusion of a random slope (V_1time_) and a slope-intercept covariance (COV01) on two degrees of freedom led to a statistically significant better model fit, suggesting that the slope of time varied across countries. The statistical inference on the added random slope is only approximate, as we do not have a restricted maximum likelihood for the logistic mixed model. Based on these results, we tested a trimmed version of the model M4 without cubic effects, model M4b. The BIC for this model was the smallest of all models.

**TABLE 3:** Model summary for random effects approach. Model comparisons are conducted on likelihood ratio test (LRT) difference test on model difference degrees of freedom, Akaike’s information criterion (AIC) and Bayes information criterion (BIC) (smaller is better). (Health behaviour in school-aged children 2006–2022).

Statistic	M0 No trend	M1 Linear	M2 Quadratic	M3 Cubic	M4 Random slope linear
Model parameters	3	4	5	6	8
AIC	1,165,085.51	1,165,032.45	1,165,020.26	1,165,020.77	1,164,981.43
BIC	1,165,120.82	1,165,079.53	1,165,079.11	1,165,091.40	1,165,075.59
Deviance	1,165,079.51	1,165,024.45	1,165,010.26	1,165,008.77	1,164,965.43
LRT		55.06	14.19	1.48	43.35
Nested model df M_k_ vs. M_k.1_ comparison		1	1	1	2
*p*-value		<0.001	<0.001	0.224	<0.001

Supplementary Table S4 show model coefficients for the selected model M4b, with a fixed part and a random part. The fixed part indicated that for the average country (at random effects = 0) there was positive linear trend component $\beta_{1}$ = 0.134 and the negative quadratic component $\beta_{2}$ = −0.125 indicated that the positive trend levelled of as a function of time.

The random intercept SD (U_0_) = 0.079 at country/region-year level, suggested that toothbrushing fluctuate within a prediction interval + −0.079*1.96 = [−0.158 to 0.158] logit units, relative to the linear slope of a country. The random intercept SD at the region-level (V_0_) = 0.421 indicated that for an average country, adolescents’ prevalence of toothbrushing would fall within the 95% prediction interval [−0.02 to 1.63], which after logit transformation to probabilities implies a prevalence of “toothbrushing more than once a day” to vary between 49% and 83% at time 0 (year 2014). The random slope of linear time (V1) with an SD of 0.122, suggested that the for the population of countries the slope of the linear component would fall within −0.134+−0.122*1.96, giving a 95% prediction interval in logit units for the linear slope of linear time [−0.11, 0.37]. The negative corelation between intercept and slope means that countries with a low level of toothbrushing tended to have a stronger positive slope of time. We also computed model-based predictions for each specific country/region, relevant for specific inference about the differences in trends. A quantile-quantile plot for each random variance component indicated a close fit to a normal distribution .for both country/region and country/region-year level, as shown in Supplementary Figures S5, S6.

Figure 2 shows the model-based predicted probability of toothbrushing as a function of time for the best fitting models of the fixed effect approach and the random effect approach. The upper half of the figure shows results from the fixed effect approach model M6 (panels A and B), and the lower half shows the results for the random effects approach model M4b (panels C and D). The confidence intervals for the average trend were notably slimmer for the fixed effect approach.

The fixed effect approach and the random effect approach predicted a group of countries and regions with a higher level of toothbrushing, and no apparent trend. For the number of regions that started with a low to medium level of toothbrushing the prediction was a trend of increased toothbrushing. The average marginal effect showed that the trend is positive but flattening.

Table 4 summarises the model information and findings from the three approaches included.

**TABLE 4:** Summary of model information, and main results by data analytic approach.

Study criterion/Approach	Information/Parameters	Main findings
Overall trend		
Stratified	Provides trends per country/region. Specific statistics on trend but no test statistic for the overall average trend	Of 35 regions. 30 regions showed a trend with a linear component, of which 27 slopes were positive. No test for overall average trend
Fixed effect	Test of overall average trend trends and the shape of trend, conditional on country/region differences	Statistically significant overall trend with linear, quadratic and cubic components
Random effect	Tests of overall trends and the shape of the trend for the average country/region	Statistically significant overall trend with linear and quadratic components
Trend differences		
Stratified	Post hoc profiles of different trend patterns, but no test statistic for the regional differences	Different slope results for countries/regions, and different types of trends across countries/regions
Fixed effect	Omnibus model tests and specific test of regional differences through contrast specification. Model-based predictions for country/region	Statistically significant country/region differences in linear, quadratic and cubic components of trend Specific test of each country/region vs. average for intercept, linear, quadratic and cubic effects
Random effect	Omnibus model tests for random effects, model-based prediction intervals for population, and specific country/region	Statistically significant country/region differences in linear slope of time Specific predictions of country/region trend differences

## Discussion

The objective of the current study was to compare the information returned from stratified, fixed effect and random effect approaches to comparative time trends in toothbrushing. The type of information and results returned from the analyses was different for the three approaches.

The stratified approach provided a high level of detail about each country/region trend, but did not provide statistical tests of direct relevance to our two research questions. To answer our research questions on the overall trend and the differences in trend we used an implicit, but non-parametric approach by counting and ranking the number of statistically significant trends, a procedure sometimes referred to as “vote counting.” Our *post hoc* classification of trend profiles indicated variation across countries and an overall upwards trend, but the count of profiles does not represent a statistical inference.

For the fixed effect approach and the random effects approach, our two research questions could be operationalised as testable hypothesis about model parameters, either expressed as fixed effects or as random effects. Both approaches provide omnibus tests as well as specific country-level inference about effects, and their conclusions overlapped but were not identical. For both approaches the omnibus tests concluded with a non-linear positive but gradually flattening trend in toothbrushing, and both approaches concluded with cross-national differences in the trends. The fixed effect approach included tests country/region by time, country/region by time quadratic, and country/region by time cubic, and provided a more detailed perspective of trend in each country/region. In studies of region differences in trends, region-specific conclusions are of key interest to the researcher, and the fixed effect approach can to high degree provide relevant information, however this level of specificity comes at the cost of model complexity. The most comprehensive models (M6 fixed effect approach), included 136 country/region contrasts, which at least from a practical perspective, is high.

The random effect approach did not include separate fixed estimates for the specific countries/regions, but produced country/region-specific conditional predictions. In a context with many countries and many time points, the specified random slope of time provide a flexible yet parsimonious approach to modelling cross-national differences in the trend. Compared to fixed effect approach, the results from the random effect approach suggested a simpler parametric shape for the overall time trend, as the cubic main effects (model M3) did not achieve statistical significance. The subtle differences in conclusion on the shape of the overall trend between the fixed effect approach and the random effects approach might reflect specification differences. The country/region-year random component (U_oj_) models random fluctuations across time, thus reducing the need for to include fixed part non-linear components for each country. Conceptually, the provision of a random country-year component can be important, by separating longer term linear trends from short term societal changes that do not follow a parametric linear curve, and therefore might reflect different underlying societal processes.

Under the current sample size and number of countries, key model assumptions were satisfied in all three approaches. However, the model assumptions of the three approaches have different sensitivity to number of country/region units included. The stratified and fixed effects approach can be conducted with 5 countries and with 35 countries without expected violations of model assumptions. For the random effects approach random variance components and standard errors of estimates tend to be downward biased when the number of higher units is small [18, 19]. Under the current frequentist approach, 35 countries or region units is just above the recommended limit of at least 30 countries to achieve accurate estimates of the logistic mixed model [18]. If the number of country units is smaller, Bayesian computation of random country-level effects is a relevant alternative as this method has less bias in small sample situations [19], but the Bayesian computation require researchers to make additional assumptions about the prior distribution.

### Limitations

We only considered polynomial specification of time. This specification may work well to capture non-linearity within a specified time frame but be less accurate in long term projections. Decisions about trend shape need to consider both the number of time points with observations and the length of the period covered. If events have occurred during the period covered, such as sudden technological innovations, macroeconomic shocks, changes in health legislation, or pandemics, a piecewise model or simple contrasts as an extension of the simple linear trend could be relevant alternative specifications to quadratic and cubic effects. In *piecewise models* the slope of a linear time effects can change at a given time point, allowing for an overall non-linear trend and turning points. Generalized additive models and generalized additive mixed models [20] provide a general regression framework for non-linear modelling of trends.

Secondly, omission of unmeasured time-invariant or time-varying independent variables might bias regression trend estimates. Unmeasured third variables at the country/region level might particularly affect the random effect approach to trend analysis, as the random region-level effect will include the effects of such unmeasured variables. For the fixed effect approach, conditioning on the main effect of region account for region level third-variables. The stratified approach might be least vulnerable to omission of region-level factors, as relevant third variables are restricted to those affecting the within-country context. As a basic strategy to minimize endogeneity, comparative time trend studies can counteract bias by collecting information on country/region indicators and include that information as covariates in the model.

Our comparison of approaches was conducted on a set of descriptive research questions, which represent an important first stage in trend analysis. Future research should examine how the three approaches can be extended to explanatory trend analysis with country-level moderators and mediators of comparative trends, where two-stage modelling [21] and hybrid random effects model [22] might provide relevant example starting points for a comparison.

### Conclusion

We compared the model information and results obtained from stratified, fixed effect, and random effect approaches to comparative trend analyses of adolescent toothbrushing. Our case clearly demonstrated that statistical inference about average time trends and trend differences is lacking with a stratified approach. For statistical inference regarding the trend and trend differences, the fixed effect approach provided a high level of specificity. The random effects approach produced similar conclusions, but with less detail and specificity in the trend for each country.
