# Supplementary material for: Methodological Approaches to Comparative Trend Analyses: The Case of Adolescent Toothbrushing
Source: Int J Public Health. 2025 Jan 10;69:1607669. doi: 10.3389/ijph.2024.1607669 (PMC11757018; doi:10.3389/ijph.2024.1607669)
Supplement: Supplementary file 1 [file DataSheet1.docx]

Supplementary table S1.

Logistic regression models of toothbrushing regressed on time in 35 countries and regions. Blockwise entry of independent variables. Time is centered on year 2014 and scaled to 10-year unit Regression coefficients are on a logit scale, reflecting change per 10-year period (Health Behaviour in School-aged Children study, 2006-2022).

|  | M1 Linear | | | | M2 Quadratic | | | | M3 Cubic | | | |
| --- | --- | --- | --- | --- | --- | --- | --- | --- | --- | --- | --- | --- |
|  | Coef | SE | t | p-value | Coef | SE | t | p-value | Coef | SE | t | p-value |
| **Austria** |  |  |  |  |  |  |  |  |  |  |  |  |
| (Intercept) | 1.14 | 0.02 | 73.12 | <0.001 | 1.15 | 0.03 | 44.86 | <0.001 | 1.15 | 0.03 | 44.73 | <0.001 |
| Time | 0.03 | 0.03 | 0.98 | 0.329 | 0.03 | 0.03 | 1.00 | 0.318 | -0.03 | 0.08 | -0.38 | 0.704 |
| Time quadratic |  |  |  |  | -0.04 | 0.06 | -0.71 | 0.475 | -0.04 | 0.06 | -0.69 | 0.493 |
| Time cubic |  |  |  |  |  |  |  |  | 0.10 | 0.14 | 0.74 | 0.460 |
| **Belgium(VLG)** |  |  |  |  |  |  |  |  |  |  |  |  |
| (Intercept) | 0.48 | 0.01 | 36.21 | <0.001 | 0.51 | 0.02 | 23.43 | <0.001 | 0.51 | 0.02 | 23.45 | <0.001 |
| Time | 0.34 | 0.02 | 15.73 | <0.001 | 0.35 | 0.02 | 15.88 | <0.001 | 0.18 | 0.08 | 2.42 | 0.016 |
| Time quadratic |  |  |  |  | -0.11 | 0.05 | -2.19 | 0.028 | -0.11 | 0.05 | -2.35 | 0.019 |
| Time cubic |  |  |  |  |  |  |  |  | 0.29 | 0.13 | 2.28 | 0.023 |
| **Belgium(WAL)** |  |  |  |  |  |  |  |  |  |  |  |  |
| (Intercept) | 0.48 | 0.01 | 36.88 | <0.001 | 0.47 | 0.02 | 23.80 | <0.001 | 0.47 | 0.02 | 23.47 | <0.001 |
| Time | 0.09 | 0.02 | 4.08 | <0.001 | 0.09 | 0.02 | 4.03 | <0.001 | 0.26 | 0.07 | 3.64 | <0.001 |
| Time quadratic |  |  |  |  | 0.03 | 0.05 | 0.54 | 0.587 | 0.04 | 0.05 | 0.75 | 0.452 |
| Time cubic |  |  |  |  |  |  |  |  | -0.30 | 0.12 | -2.47 | 0.014 |
| **Canada** |  |  |  |  |  |  |  |  |  |  |  |  |
| (Intercept) | 0.71 | 0.01 | 80.33 | <0.001 | 0.74 | 0.01 | 57.24 | <0.001 | 0.74 | 0.01 | 57.21 | <0.001 |
| Time | -0.14 | 0.02 | -8.35 | <0.001 | -0.12 | 0.02 | -7.00 | <0.001 | 0.02 | 0.04 | 0.38 | 0.705 |
| Time quadratic |  |  |  |  | -0.13 | 0.04 | -3.72 | <0.001 | -0.11 | 0.04 | -3.11 | 0.002 |
| Time cubic |  |  |  |  |  |  |  |  | -0.28 | 0.08 | -3.53 | <0.001 |
| **Switzerland** |  |  |  |  |  |  |  |  |  |  |  |  |
| (Intercept) | 1.70 | 0.02 | 109.02 | <0.001 | 1.78 | 0.02 | 74.46 | <0.001 | 1.77 | 0.02 | 74.19 | <0.001 |
| Time | -0.07 | 0.03 | -2.56 | 0.011 | -0.05 | 0.03 | -1.89 | 0.059 | 0.23 | 0.08 | 2.91 | 0.004 |
| Time quadratic |  |  |  |  | -0.28 | 0.06 | -4.71 | <0.001 | -0.25 | 0.06 | -4.27 | <0.001 |
| Time cubic |  |  |  |  |  |  |  |  | -0.53 | 0.14 | -3.82 | <0.001 |
| **Czech republic** |  |  |  |  |  |  |  |  |  |  |  |  |
| (Intercept) | 0.98 | 0.01 | 78.87 | <0.001 | 0.95 | 0.02 | 49.07 | <0.001 | 0.93 | 0.02 | 45.76 | <0.001 |
| Time | 0.12 | 0.02 | 5.63 | <0.001 | 0.11 | 0.02 | 5.05 | <0.001 | 0.24 | 0.06 | 3.81 | <0.001 |
| Time quadratic |  |  |  |  | 0.09 | 0.04 | 2.12 | 0.034 | 0.13 | 0.05 | 2.72 | 0.007 |
| Time cubic |  |  |  |  |  |  |  |  | -0.24 | 0.11 | -2.23 | 0.026 |
| **Germany** |  |  |  |  |  |  |  |  |  |  |  |  |
| (Intercept) | 1.31 | 0.01 | 90.09 | <0.001 | 1.39 | 0.02 | 58.43 | <0.001 | 1.39 | 0.02 | 58.37 | <0.001 |
| Time | -0.03 | 0.02 | -1.07 | 0.285 | -0.03 | 0.02 | -1.28 | 0.202 | 0.00 | 0.09 | -0.04 | 0.971 |
| Time quadratic |  |  |  |  | -0.23 | 0.05 | -4.37 | <0.001 | -0.23 | 0.05 | -4.38 | <0.001 |
| Time cubic |  |  |  |  |  |  |  |  | -0.05 | 0.14 | -0.34 | 0.736 |
| **Denmark** |  |  |  |  |  |  |  |  |  |  |  |  |
| (Intercept) | 1.27 | 0.02 | 78.23 | <0.001 | 1.26 | 0.03 | 46.46 | <0.001 | 1.28 | 0.03 | 46.38 | <0.001 |
| Time | 0.10 | 0.03 | 3.62 | <0.001 | 0.10 | 0.03 | 3.63 | <0.001 | 0.52 | 0.10 | 5.45 | <0.001 |
| Time quadratic |  |  |  |  | 0.03 | 0.06 | 0.50 | 0.620 | 0.00 | 0.06 | 0.00 | 0.998 |
| Time cubic |  |  |  |  |  |  |  |  | -0.74 | 0.16 | -4.62 | <0.001 |
| **Estonia** |  |  |  |  |  |  |  |  |  |  |  |  |
| (Intercept) | 0.57 | 0.01 | 41.02 | <0.001 | 0.56 | 0.02 | 25.32 | <0.001 | 0.56 | 0.02 | 25.32 | <0.001 |
| Time | 0.05 | 0.02 | 2.19 | 0.029 | 0.05 | 0.02 | 2.18 | 0.029 | 0.01 | 0.07 | 0.18 | 0.854 |
| Time quadratic |  |  |  |  | 0.03 | 0.05 | 0.62 | 0.533 | 0.03 | 0.05 | 0.61 | 0.544 |
| Time cubic |  |  |  |  |  |  |  |  | 0.07 | 0.13 | 0.56 | 0.574 |
| **Spain** |  |  |  |  |  |  |  |  |  |  |  |  |
| (Intercept) | 0.64 | 0.01 | 53.28 | <0.001 | 0.68 | 0.02 | 40.49 | <0.001 | 0.68 | 0.02 | 40.51 | <0.001 |
| Time | 0.21 | 0.02 | 9.41 | <0.001 | 0.18 | 0.02 | 8.02 | <0.001 | 0.33 | 0.07 | 4.56 | <0.001 |
| Time quadratic |  |  |  |  | -0.15 | 0.04 | -3.43 | 0.001 | -0.16 | 0.04 | -3.74 | <0.001 |
| Time cubic |  |  |  |  |  |  |  |  | -0.27 | 0.13 | -2.16 | 0.031 |
| **Finland** |  |  |  |  |  |  |  |  |  |  |  |  |
| (Intercept) | 0.34 | 0.01 | 25.33 | <0.001 | 0.44 | 0.02 | 22.55 | <0.001 | 0.44 | 0.02 | 21.91 | <0.001 |
| Time | 0.44 | 0.03 | 17.67 | <0.001 | 0.42 | 0.03 | 16.83 | <0.001 | 0.44 | 0.07 | 6.22 | <0.001 |
| Time quadratic |  |  |  |  | -0.35 | 0.05 | -6.98 | <0.001 | -0.35 | 0.05 | -6.91 | <0.001 |
| Time cubic |  |  |  |  |  |  |  |  | -0.04 | 0.12 | -0.34 | 0.731 |
| **France** |  |  |  |  |  |  |  |  |  |  |  |  |
| (Intercept) | 1.00 | 0.01 | 80.24 | <0.001 | 1.13 | 0.02 | 56.59 | <0.001 | 1.12 | 0.02 | 56.28 | <0.001 |
| Time | 0.30 | 0.02 | 13.72 | <0.001 | 0.27 | 0.02 | 12.06 | <0.001 | 0.53 | 0.06 | 8.31 | <0.001 |
| Time quadratic |  |  |  |  | -0.41 | 0.05 | -8.50 | <0.001 | -0.42 | 0.05 | -8.68 | <0.001 |
| Time cubic |  |  |  |  |  |  |  |  | -0.48 | 0.11 | -4.36 | <0.001 |
| **England** |  |  |  |  |  |  |  |  |  |  |  |  |
| (Intercept) | 1.21 | 0.02 | 73.36 | <0.001 | 1.27 | 0.03 | 49.73 | <0.001 | 1.27 | 0.03 | 49.68 | <0.001 |
| Time | 0.08 | 0.03 | 2.90 | 0.004 | 0.08 | 0.03 | 2.74 | 0.006 | -0.02 | 0.10 | -0.22 | 0.825 |
| Time quadratic |  |  |  |  | -0.19 | 0.06 | -3.20 | 0.001 | -0.19 | 0.06 | -3.15 | 0.002 |
| Time cubic |  |  |  |  |  |  |  |  | 0.18 | 0.17 | 1.07 | 0.286 |
| **Scotland** |  |  |  |  |  |  |  |  |  |  |  |  |
| (Intercept) | 1.09 | 0.01 | 78.17 | <0.001 | 1.20 | 0.02 | 56.24 | <0.001 | 1.21 | 0.02 | 56.02 | <0.001 |
| Time | 0.06 | 0.03 | 2.50 | 0.012 | 0.04 | 0.02 | 1.74 | 0.081 | 0.20 | 0.07 | 2.69 | 0.007 |
| Time quadratic |  |  |  |  | -0.38 | 0.05 | -7.32 | <0.001 | -0.39 | 0.05 | -7.54 | <0.001 |
| Time cubic |  |  |  |  |  |  |  |  | -0.28 | 0.13 | -2.24 | 0.025 |
| **Wales** |  |  |  |  |  |  |  |  |  |  |  |  |
| (Intercept) | 0.95 | 0.01 | 81.69 | <0.001 | 1.03 | 0.02 | 58.75 | <0.001 | 1.01 | 0.02 | 52.80 | <0.001 |
| Time | -0.02 | 0.02 | -1.22 | 0.223 | 0.04 | 0.02 | 1.88 | 0.060 | 0.14 | 0.06 | 2.52 | 0.012 |
| Time quadratic |  |  |  |  | -0.25 | 0.04 | -6.34 | <0.001 | -0.20 | 0.04 | -4.62 | <0.001 |
| Time cubic |  |  |  |  |  |  |  |  | -0.19 | 0.10 | -1.99 | 0.046 |
| **Greenland** |  |  |  |  |  |  |  |  |  |  |  |  |
| (Intercept) | 0.39 | 0.03 | 14.40 | <0.001 | 0.54 | 0.04 | 12.29 | <0.001 | 0.54 | 0.04 | 12.25 | <0.001 |
| Time | 0.30 | 0.05 | 6.34 | <0.001 | 0.28 | 0.05 | 6.00 | <0.001 | 0.01 | 0.14 | 0.10 | 0.919 |
| Time quadratic |  |  |  |  | -0.44 | 0.10 | -4.37 | <0.001 | -0.43 | 0.10 | -4.27 | <0.001 |
| Time cubic |  |  |  |  |  |  |  |  | 0.48 | 0.24 | 1.97 | 0.049 |
| **Greece** |  |  |  |  |  |  |  |  |  |  |  |  |
| (Intercept) | 0.01 | 0.01 | 0.93 | 0.350 | 0.02 | 0.02 | 0.88 | 0.378 | 0.02 | 0.02 | 0.70 | 0.481 |
| Time | 0.30 | 0.02 | 13.15 | <0.001 | 0.30 | 0.02 | 12.99 | <0.001 | 0.10 | 0.07 | 1.43 | 0.154 |
| Time quadratic |  |  |  |  | -0.02 | 0.05 | -0.38 | 0.703 | -0.02 | 0.05 | -0.49 | 0.623 |
| Time cubic |  |  |  |  |  |  |  |  | 0.37 | 0.12 | 2.96 | 0.003 |
| **Croatia** |  |  |  |  |  |  |  |  |  |  |  |  |
| (Intercept) | 0.53 | 0.01 | 41.56 | <0.001 | 0.55 | 0.02 | 28.48 | <0.001 | 0.55 | 0.02 | 28.31 | <0.001 |
| Time | 0.24 | 0.02 | 10.56 | <0.001 | 0.24 | 0.02 | 10.59 | <0.001 | 0.18 | 0.07 | 2.66 | 0.008 |
| Time quadratic |  |  |  |  | -0.09 | 0.05 | -1.79 | 0.074 | -0.08 | 0.05 | -1.73 | 0.084 |
| Time cubic |  |  |  |  |  |  |  |  | 0.12 | 0.12 | 1.07 | 0.284 |
| **Hungary** |  |  |  |  |  |  |  |  |  |  |  |  |
| (Intercept) | 0.38 | 0.01 | 26.55 | <0.001 | 0.36 | 0.02 | 16.37 | <0.001 | 0.36 | 0.02 | 16.27 | <0.001 |
| Time | 0.06 | 0.03 | 2.33 | 0.020 | 0.06 | 0.03 | 2.26 | 0.024 | 0.02 | 0.07 | 0.23 | 0.821 |
| Time quadratic |  |  |  |  | 0.06 | 0.06 | 1.18 | 0.238 | 0.07 | 0.06 | 1.20 | 0.232 |
| Time cubic |  |  |  |  |  |  |  |  | 0.08 | 0.13 | 0.61 | 0.543 |
| **Ireland** |  |  |  |  |  |  |  |  |  |  |  |  |
| (Intercept) | 0.71 | 0.01 | 47.94 | <0.001 | 0.78 | 0.02 | 33.60 | <0.001 | 0.78 | 0.02 | 33.35 | <0.001 |
| Time | 0.28 | 0.03 | 10.50 | <0.001 | 0.26 | 0.03 | 10.03 | <0.001 | 0.20 | 0.08 | 2.55 | 0.011 |
| Time quadratic |  |  |  |  | -0.20 | 0.06 | -3.67 | <0.001 | -0.20 | 0.06 | -3.54 | <0.001 |
| Time cubic |  |  |  |  |  |  |  |  | 0.12 | 0.14 | 0.86 | 0.391 |
| **Israel** |  |  |  |  |  |  |  |  |  |  |  |  |
| (Intercept) | 0.70 | 0.02 | 46.38 | <0.001 | 0.74 | 0.03 | 28.49 | <0.001 | 0.76 | 0.03 | 28.63 | <0.001 |
| Time | 0.40 | 0.02 | 16.54 | <0.001 | 0.40 | 0.02 | 16.45 | <0.001 | 0.76 | 0.09 | 8.67 | <0.001 |
| Time quadratic |  |  |  |  | -0.10 | 0.06 | -1.79 | 0.073 | -0.14 | 0.06 | -2.42 | 0.015 |
| Time cubic |  |  |  |  |  |  |  |  | -0.62 | 0.15 | -4.25 | <0.001 |
| **Iceland** |  |  |  |  |  |  |  |  |  |  |  |  |
| (Intercept) | 0.77 | 0.01 | 77.46 | <0.001 | 0.84 | 0.02 | 54.54 | <0.001 | 0.83 | 0.02 | 53.66 | <0.001 |
| Time | 0.18 | 0.02 | 9.99 | <0.001 | 0.18 | 0.02 | 10.14 | <0.001 | 0.08 | 0.05 | 1.38 | 0.168 |
| Time quadratic |  |  |  |  | -0.22 | 0.04 | -6.01 | <0.001 | -0.21 | 0.04 | -5.80 | <0.001 |
| Time cubic |  |  |  |  |  |  |  |  | 0.18 | 0.09 | 1.95 | 0.052 |
| **Italy** |  |  |  |  |  |  |  |  |  |  |  |  |
| (Intercept) | 1.04 | 0.02 | 66.92 | <0.001 | 1.05 | 0.02 | 43.07 | <0.001 | 1.05 | 0.02 | 43.14 | <0.001 |
| Time | 0.06 | 0.03 | 2.25 | 0.025 | 0.06 | 0.03 | 2.27 | 0.023 | 0.36 | 0.08 | 4.46 | <0.001 |
| Time quadratic |  |  |  |  | -0.03 | 0.06 | -0.47 | 0.640 | -0.03 | 0.06 | -0.54 | 0.591 |
| Time cubic |  |  |  |  |  |  |  |  | -0.56 | 0.14 | -3.93 | <0.001 |
| **Lithuania** |  |  |  |  |  |  |  |  |  |  |  |  |
| (Intercept) | -0.01 | 0.01 | -0.46 | 0.648 | 0.03 | 0.02 | 1.73 | 0.084 | 0.02 | 0.02 | 1.17 | 0.242 |
| Time | 0.30 | 0.02 | 13.42 | <0.001 | 0.30 | 0.02 | 13.37 | <0.001 | -0.04 | 0.07 | -0.62 | 0.534 |
| Time quadratic |  |  |  |  | -0.12 | 0.05 | -2.66 | 0.008 | -0.11 | 0.05 | -2.27 | 0.024 |
| Time cubic |  |  |  |  |  |  |  |  | 0.62 | 0.12 | 5.08 | <0.001 |
| **Luxembourg** |  |  |  |  |  |  |  |  |  |  |  |  |
| (Intercept) | 0.89 | 0.02 | 57.18 | <0.001 | 0.95 | 0.03 | 37.45 | <0.001 | 0.95 | 0.03 | 37.43 | <0.001 |
| Time | 0.05 | 0.03 | 2.01 | 0.045 | 0.05 | 0.03 | 1.96 | 0.050 | -0.08 | 0.08 | -0.93 | 0.350 |
| Time quadratic |  |  |  |  | -0.18 | 0.06 | -3.13 | 0.002 | -0.18 | 0.06 | -3.09 | 0.002 |
| Time cubic |  |  |  |  |  |  |  |  | 0.23 | 0.14 | 1.66 | 0.097 |
| **Latvia** |  |  |  |  |  |  |  |  |  |  |  |  |
| (Intercept) | 0.11 | 0.01 | 8.39 | <0.001 | 0.06 | 0.02 | 3.26 | 0.001 | 0.06 | 0.02 | 3.24 | 0.001 |
| Time | 0.08 | 0.02 | 3.62 | <0.001 | 0.08 | 0.02 | 3.28 | 0.001 | 0.17 | 0.07 | 2.41 | 0.016 |
| Time quadratic |  |  |  |  | 0.14 | 0.05 | 2.87 | 0.004 | 0.14 | 0.05 | 2.95 | 0.003 |
| Time cubic |  |  |  |  |  |  |  |  | -0.18 | 0.12 | -1.45 | 0.148 |
| **North Macedonia** |  |  |  |  |  |  |  |  |  |  |  |  |
| (Intercept) | 0.55 | 0.01 | 38.94 | <0.001 | 0.58 | 0.02 | 25.99 | <0.001 | 0.58 | 0.02 | 26.04 | <0.001 |
| Time | 0.10 | 0.02 | 4.26 | <0.001 | 0.10 | 0.02 | 4.02 | <0.001 | -0.05 | 0.08 | -0.62 | 0.535 |
| Time quadratic |  |  |  |  | -0.10 | 0.05 | -1.97 | 0.049 | -0.10 | 0.05 | -1.93 | 0.054 |
| Time cubic |  |  |  |  |  |  |  |  | 0.26 | 0.13 | 2.02 | 0.043 |
| **Netherlands** |  |  |  |  |  |  |  |  |  |  |  |  |
| (Intercept) | 1.26 | 0.02 | 77.68 | <0.001 | 1.29 | 0.03 | 50.80 | <0.001 | 1.29 | 0.03 | 50.79 | <0.001 |
| Time | -0.02 | 0.03 | -0.56 | 0.573 | -0.02 | 0.03 | -0.56 | 0.575 | 0.11 | 0.08 | 1.34 | 0.179 |
| Time quadratic |  |  |  |  | -0.09 | 0.06 | -1.42 | 0.156 | -0.09 | 0.06 | -1.40 | 0.160 |
| Time cubic |  |  |  |  |  |  |  |  | -0.24 | 0.15 | -1.63 | 0.103 |
| **Norway** |  |  |  |  |  |  |  |  |  |  |  |  |
| (Intercept) | 1.10 | 0.02 | 63.82 | <0.001 | 1.13 | 0.03 | 40.88 | <0.001 | 1.15 | 0.03 | 40.86 | <0.001 |
| Time | -0.06 | 0.03 | -2.06 | 0.040 | -0.06 | 0.03 | -2.17 | 0.030 | 0.26 | 0.09 | 2.79 | 0.005 |
| Time quadratic |  |  |  |  | -0.09 | 0.06 | -1.44 | 0.151 | -0.12 | 0.06 | -1.87 | 0.062 |
| Time cubic |  |  |  |  |  |  |  |  | -0.58 | 0.16 | -3.67 | <0.001 |
| **Poland** |  |  |  |  |  |  |  |  |  |  |  |  |
| (Intercept) | 0.65 | 0.01 | 48.71 | <0.001 | 0.74 | 0.02 | 34.14 | <0.001 | 0.74 | 0.02 | 33.85 | <0.001 |
| Time | -0.04 | 0.02 | -1.94 | 0.052 | -0.05 | 0.02 | -2.07 | 0.039 | 0.23 | 0.07 | 3.18 | 0.002 |
| Time quadratic |  |  |  |  | -0.27 | 0.05 | -5.38 | <0.001 | -0.26 | 0.05 | -5.23 | <0.001 |
| Time cubic |  |  |  |  |  |  |  |  | -0.50 | 0.13 | -4.02 | <0.001 |
| **Portugal** |  |  |  |  |  |  |  |  |  |  |  |  |
| (Intercept) | 0.77 | 0.01 | 54.82 | <0.001 | 0.81 | 0.02 | 37.75 | <0.001 | 0.82 | 0.02 | 37.83 | <0.001 |
| Time | 0.23 | 0.03 | 9.08 | <0.001 | 0.23 | 0.03 | 9.20 | <0.001 | 0.01 | 0.07 | 0.14 | 0.892 |
| Time quadratic |  |  |  |  | -0.14 | 0.05 | -2.72 | 0.007 | -0.16 | 0.05 | -2.96 | 0.003 |
| Time cubic |  |  |  |  |  |  |  |  | 0.41 | 0.13 | 3.20 | 0.001 |
| **Romania** |  |  |  |  |  |  |  |  |  |  |  |  |
| (Intercept) | -0.03 | 0.01 | -2.73 | 0.006 | -0.08 | 0.02 | -3.80 | <0.001 | -0.08 | 0.02 | -3.70 | <0.001 |
| Time | 0.16 | 0.02 | 7.55 | <0.001 | 0.14 | 0.02 | 6.91 | <0.001 | 0.31 | 0.07 | 4.55 | <0.001 |
| Time quadratic |  |  |  |  | 0.13 | 0.05 | 2.71 | 0.007 | 0.13 | 0.05 | 2.81 | 0.005 |
| Time cubic |  |  |  |  |  |  |  |  | -0.29 | 0.12 | -2.54 | 0.011 |
| **Sweden** |  |  |  |  |  |  |  |  |  |  |  |  |
| (Intercept) | 1.55 | 0.02 | 96.53 | <0.001 | 1.52 | 0.02 | 66.87 | <0.001 | 1.52 | 0.02 | 66.20 | <0.001 |
| Time | 0.10 | 0.03 | 3.15 | 0.002 | 0.10 | 0.03 | 3.13 | 0.002 | 0.19 | 0.09 | 2.22 | 0.026 |
| Time quadratic |  |  |  |  | 0.11 | 0.06 | 1.82 | 0.070 | 0.11 | 0.06 | 1.72 | 0.086 |
| Time cubic |  |  |  |  |  |  |  |  | -0.18 | 0.15 | -1.17 | 0.244 |
| **Slovenia** |  |  |  |  |  |  |  |  |  |  |  |  |
| (Intercept) | 0.74 | 0.01 | 57.00 | <0.001 | 0.81 | 0.02 | 38.87 | <0.001 | 0.81 | 0.02 | 38.87 | <0.001 |
| Time | 0.33 | 0.02 | 14.69 | <0.001 | 0.33 | 0.02 | 14.86 | <0.001 | 0.40 | 0.07 | 5.83 | <0.001 |
| Time quadratic |  |  |  |  | -0.20 | 0.05 | -4.16 | <0.001 | -0.20 | 0.05 | -4.17 | <0.001 |
| Time cubic |  |  |  |  |  |  |  |  | -0.12 | 0.12 | -1.04 | 0.297 |
| **Slovakia** |  |  |  |  |  |  |  |  |  |  |  |  |
| (Intercept) | 0.45 | 0.01 | 35.15 | <0.001 | 0.53 | 0.02 | 27.15 | <0.001 | 0.53 | 0.02 | 27.17 | <0.001 |
| Time | 0.09 | 0.02 | 3.76 | <0.001 | 0.10 | 0.02 | 4.35 | <0.001 | 0.20 | 0.07 | 2.87 | 0.004 |
| Time quadratic |  |  |  |  | -0.24 | 0.05 | -5.06 | <0.001 | -0.24 | 0.05 | -5.01 | <0.001 |
| Time cubic |  |  |  |  |  |  |  |  | -0.18 | 0.12 | -1.47 | 0.141 |

Supplementary Table S2.

Model summary statistics for stratified approach logistic regression of toothbrushing regressed on time. Numbers in bold represent best fitting model according to summary statistic. Likelihood ratio test (LRT) statistic is tested on 1 degree of freedom, M1 vs null model, M2 vs M1, M3 vs M2 (Health Behaviour in School-aged Children study, 2006-2022).

| Region/Statistic | M1 Linear | M2  Quadratic | M3  Cubic |
| --- | --- | --- | --- |
| Austria |  |  |  |
| Deviance | 24871.48 | 24870.97 | 24870.43 |
| AIC | 24875.48 | 24876.97 | 24878.43 |
| BIC | 24891.52 | 24901.03 | 24910.50 |
| LRT | 0.95 | 0.51 | 0.55 |
| Belgium (VLG) |  |  |  |
| Deviance | 34056.16 | 34051.34 | **34046.14** |
| AIC | 34060.16 | 34057.34 | **34054.14** |
| BIC | **34076.49** | 34081.84 | 34086.80 |
| LRT | 248.39 | 4.81 | 5.20 |
| Belgium (WAL) |  |  |  |
| Deviance | 33836.56 | 33836.26 | **33830.17** |
| AIC | 33840.56 | 33842.26 | **33838.17** |
| BIC | **33856.85** | 33866.70 | 33870.75 |
| LRT | 16.61 | 0.29 | 6.09 |
| Canada |  |  |  |
| Deviance | 76016.75 | 76002.99 | **75990.48** |
| AIC | 76020.75 | 76008.99 | **75998.48** |
| BIC | 76038.75 | 76035.98 | **76034.48** |
| LRT | 69.84 | 13.77 | 12.51 |
| Switzerland |  |  |  |
| Deviance | 27823.43 | 27801.41 | **27786.79** |
| AIC | 27827.43 | 27807.41 | **27794.79** |
| BIC | 27844.19 | 27832.55 | **27828.30** |
| LRT | 6.54 | 22.02 | 14.63 |
| Czech Republic |  |  |  |
| Deviance | 44817.04 | 44812.52 | **44807.54** |
| AIC | 44821.04 | 44818.52 | **44815.54** |
| BIC | **44838.16** | 44844.20 | 44849.78 |
| LRT | 31.55 | 4.51 | 4.98 |
| Germany |  |  |  |
| Deviance | 29385.12 | **29366.04** | 29365.92 |
| AIC | 29389.12 | **29372.04** | 29373.92 |
| BIC | 29405.63 | **29396.80** | 29406.94 |
| LRT | 1.14 | 19.09 | 0.11 |
| Denmark |  |  |  |
| Deviance | 23362.57 | 23362.32 | **23340.92** |
| AIC | 23366.57 | 23368.32 | **23348.92** |
| BIC | 23382.58 | 23392.35 | **23380.95** |
| LRT | 13.12 | 0.25 | 21.40 |
| Estonia |  |  |  |
| Deviance | **28979.28** | 28978.89 | 28978.57 |
| AIC | **28983.28** | 28984.89 | 28986.57 |
| BIC | **28999.29** | 29008.91 | 29018.60 |
| LRT | 4.80 | 0.39 | 0.32 |
| Spain |  |  |  |
| Deviance | 42762.42 | 42750.67 | **42746.02** |
| AIC | 42766.42 | 42756.67 | **42754.02** |
| BIC | 42783.23 | **42781.89** | 42787.64 |
| LRT | 88.97 | 11.75 | 4.65 |
| Finland |  |  |  |
| Deviance | 32995.14 | **32946.52** | 32946.40 |
| AIC | 32999.14 | **32952.52** | 32954.40 |
| BIC | 33015.35 | **32976.82** | 32986.80 |
| LRT | 317.94 | 48.63 | 0.12 |
| France |  |  |  |
| Deviance | 38522.18 | 38450.31 | **38431.36** |
| AIC | 38526.18 | 38456.31 | **38439.36** |
| BIC | 38543.00 | 38481.54 | **38473.00** |
| LRT | 189.20 | 71.87 | 18.95 |
| England |  |  |  |
| Deviance | 22542.56 | **22532.33** | 22531.19 |
| AIC | 22546.56 | **22538.33** | 22539.19 |
| BIC | 22562.46 | **22562.17** | 22570.98 |
| LRT | 8.44 | 10.23 | 1.14 |
| Scotland |  |  |  |
| Deviance | 31769.94 | 31716.53 | **31711.53** |
| AIC | 31773.94 | 31722.53 | **31719.53** |
| BIC | 31790.42 | **31747.26** | 31752.50 |
| LRT | 6.27 | 53.41 | 5.00 |
| Wales |  |  |  |
| Deviance | 77954.77 | 77914.63 | **77910.65** |
| AIC | 77958.77 | 77920.63 | **77918.65** |
| BIC | 77976.95 | **77947.90** | 77955.02 |
| LRT | 1.49 | 40.14 | 3.97 |
| Greenland |  |  |  |
| Deviance | 7767.69 | 7748.55 | **7744.67** |
| AIC | 7771.69 | 7754.55 | **7752.67** |
| BIC | 7785.01 | **7774.53** | 7779.31 |
| LRT | 40.48 | 19.14 | 3.88 |
| Greece |  |  |  |
| Deviance | 31431.41 | 31431.26 | **31422.49** |
| AIC | 31435.41 | 31437.26 | **31430.49** |
| BIC | **31451.48** | 31461.37 | 31462.63 |
| LRT | 174.10 | 0.15 | 8.77 |
| Croatia |  |  |  |
| Deviance | **35501.52** | 35498.33 | 35497.18 |
| AIC | 35505.52 | **35504.33** | 35505.18 |
| BIC | **35521.92** | 35528.93 | 35537.99 |
| LRT | 112.05 | 3.19 | 1.15 |
| Hungary |  |  |  |
| Deviance | **26880.85** | 26879.46 | 26879.09 |
| AIC | **26884.85** | 26885.46 | 26887.09 |
| BIC | **26900.65** | 26909.15 | 26918.68 |
| LRT | 5.43 | 1.39 | 0.37 |
| Ireland |  |  |  |
| Deviance | 26631.84 | **26618.40** | 26617.66 |
| AIC | 26635.84 | **26624.40** | 26625.66 |
| BIC | 26651.75 | **26648.25** | 26657.47 |
| LRT | 111.21 | 13.44 | 0.74 |
| Israel |  |  |  |
| Deviance | 26000.04 | 25996.83 | **25978.66** |
| AIC | 26004.04 | 26002.83 | **25986.66** |
| BIC | 26019.90 | 26026.61 | **26018.38** |
| LRT | 278.20 | 3.22 | 18.17 |
| Iceland |  |  |  |
| Deviance | 59565.45 | **59529.32** | 59525.54 |
| AIC | 59569.45 | 59535.32 | **59533.54** |
| BIC | 59586.99 | **59561.63** | 59568.62 |
| LRT | 100.28 | 36.13 | 3.78 |
| Italy |  |  |  |
| Deviance | 24702.58 | 24702.36 | **24686.89** |
| AIC | 24706.58 | 24708.36 | **24694.89** |
| BIC | **24722.53** | 24732.29 | 24726.79 |
| LRT | 5.05 | 0.22 | 15.47 |
| Lithuania |  |  |  |
| Deviance | 34885.50 | 34878.42 | **34852.65** |
| AIC | 34889.50 | 34884.42 | **34860.65** |
| BIC | 34905.78 | 34908.84 | **34893.20** |
| LRT | 181.10 | 7.08 | 25.78 |
| Luxembourg |  |  |  |
| Deviance | 24233.71 | **24223.94** | 24221.19 |
| AIC | 24237.71 | 24229.94 | **24229.19** |
| BIC | **24253.52** | 24253.67 | 24260.82 |
| LRT | 4.03 | 9.76 | 2.75 |
| Latvia |  |  |  |
| Deviance | 33539.71 | **33531.46** | 33529.36 |
| AIC | 33543.71 | 33537.46 | **33537.36** |
| BIC | **33559.91** | 33561.74 | 33569.75 |
| LRT | 13.10 | 8.26 | 2.09 |
| North Macedonia |  |  |  |
| Deviance | 29006.16 | 29002.27 | **28998.19** |
| AIC | 29010.16 | 29008.27 | **29006.19** |
| BIC | **29026.16** | 29032.27 | 29038.19 |
| LRT | 18.13 | 3.89 | 4.08 |
| Netherlands |  |  |  |
| Deviance | 23367.20 | 23365.19 | 23362.53 |
| AIC | 23371.20 | 23371.19 | 23370.53 |
| BIC | 23387.21 | 23395.20 | 23402.55 |
| LRT | 0.32 | 2.01 | 2.66 |
| Norway |  |  |  |
| Deviance | 20591.81 | 20589.75 | **20576.23** |
| AIC | 20595.81 | 20595.75 | **20584.23** |
| BIC | **20611.45** | 20619.20 | 20615.50 |
| LRT | 4.23 | 2.07 | 13.52 |
| Poland |  |  |  |
| Deviance | 31884.32 | 31855.39 | **31839.23** |
| AIC | 31888.32 | 31861.39 | **31847.23** |
| BIC | 31904.56 | 31885.74 | **31879.71** |
| LRT | 3.77 | 28.94 | 16.15 |
| Portugal |  |  |  |
| Deviance | 29652.14 | 29644.77 | **29634.56** |
| AIC | 29656.14 | 29650.77 | **29642.56** |
| BIC | 29672.30 | 29675.02 | **29674.88** |
| LRT | 82.50 | 7.37 | 10.21 |
| Romania |  |  |  |
| Deviance | 36600.75 | 36593.43 | **36586.98** |
| AIC | 36604.75 | 36599.43 | **36594.98** |
| BIC | **36621.11** | 36623.98 | 36627.71 |
| LRT | 57.03 | 7.32 | 6.45 |
| Sweden |  |  |  |
| Deviance | **25186.35** | 25183.05 | 25181.68 |
| AIC | 25190.35 | **25189.05** | 25189.68 |
| BIC | **25206.77** | 25213.67 | 25222.51 |
| LRT | 9.93 | 3.31 | 1.36 |
| Slovenia |  |  |  |
| Deviance | 34273.55 | **34256.23** | 34255.14 |
| AIC | 34277.55 | **34262.23** | 34263.14 |
| BIC | 34293.99 | **34286.89** | 34296.02 |
| LRT | 217.25 | 17.32 | 1.09 |
| Slovakia |  |  |  |
| Deviance | 33816.46 | **33790.91** | 33788.75 |
| AIC | 33820.46 | 33796.91 | **33796.75** |
| BIC | 33836.74 | **33821.33** | 33829.31 |
| LRT | 14.14 | 25.55 | 2.16 |

Supplementary table S3.

Logistic regression model of toothbrushing regressed on main and interaction terms of time and region, fixed effect approach, model M6. Country/region is deviation coded, to express effects as deviation from the mean. Time is centered on year 2014, and scaled to 10-year unit. Regression coefficients for time are on a logit scale, reflecting change per 10-year period (Health Behaviour in School-aged Children, 2006-2022).

|  | Coef | SE | 95% CI | z | p-value |
| --- | --- | --- | --- | --- | --- |
| Intercept | 0.79 | 3.88E-03 | [ 0.79, 0.80] | 204.65 | < .001 |
| Time Linear | 0.19 | 0.01 | [ 0.16, 0.21] | 14.01 | < .001 |
| Time Quadratic | -0.13 | 9.15E-03 | [-0.14, -0.11] | -13.72 | < .001 |
| Time Cubic | -0.10 | 0.02 | [-0.14, -0.05] | -4.22 | < .001 |
| **County/Region** |  |  |  |  |  |
| Austria | 0.36 | 0.03 | [ 0.31, 0.41] | 14.11 | < .001 |
| Belgium(VLG) | -0.28 | 0.02 | [-0.32, -0.24] | -12.99 | < .001 |
| Belgium(WAL) | -0.33 | 0.02 | [-0.37, -0.29] | -16.71 | < .001 |
| Canada | -0.05 | 0.01 | [-0.08, -0.03] | -4.08 | < .001 |
| Switzerland | 0.98 | 0.02 | [ 0.93, 1.03] | 41.59 | < .001 |
| Czech Republic | 0.14 | 0.02 | [ 0.10, 0.18] | 6.84 | < .001 |
| Germany | 0.60 | 0.02 | [ 0.55, 0.64] | 25.41 | < .001 |
| Denmark | 0.49 | 0.03 | [ 0.43, 0.54] | 17.96 | < .001 |
| Estonia | -0.23 | 0.02 | [-0.27, -0.19] | -10.52 | < .001 |
| Spain | -0.11 | 0.02 | [-0.15, -0.08] | -6.83 | < .001 |
| Finland | -0.35 | 0.02 | [-0.39, -0.32] | -17.83 | < .001 |
| France | 0.33 | 0.02 | [ 0.29, 0.37] | 16.63 | < .001 |
| England | 0.47 | 0.03 | [ 0.43, 0.52] | 18.9 | < .001 |
| Scotland | 0.41 | 0.02 | [ 0.37, 0.46] | 19.42 | < .001 |
| Wales | 0.22 | 0.02 | [ 0.18, 0.26] | 11.5 | < .001 |
| Greenland | -0.26 | 0.04 | [-0.34, -0.18] | -6.08 | < .001 |
| Greece | -0.78 | 0.02 | [-0.82, -0.74] | -36.86 | < .001 |
| Croatia | -0.25 | 0.02 | [-0.28, -0.21] | -12.74 | < .001 |
| Hungary | -0.43 | 0.02 | [-0.48, -0.39] | -19.7 | < .001 |
| Ireland | -0.02 | 0.02 | [-0.06, 0.03] | -0.82 | 0.414 |
| Israel | -0.04 | 0.03 | [-0.09, 0.01] | -1.53 | 0.127 |
| Iceland | 0.04 | 0.02 | [ 0.01, 0.07] | 2.29 | 0.022 |
| Italy | 0.26 | 0.02 | [ 0.21, 0.31] | 10.76 | < .001 |
| Lithuania | -0.77 | 0.02 | [-0.81, -0.73] | -39.69 | < .001 |
| Luxembourg | 0.15 | 0.02 | [ 0.11, 0.20] | 6.21 | < .001 |
| Latvia | -0.73 | 0.02 | [-0.77, -0.69] | -37.05 | < .001 |
| North Macedonia | -0.21 | 0.02 | [-0.26, -0.17] | -9.72 | < .001 |
| Netherlands | 0.49 | 0.02 | [ 0.44, 0.54] | 19.73 | < .001 |
| Norway | 0.35 | 0.03 | [ 0.30, 0.40] | 12.75 | < .001 |
| Poland | -0.06 | 0.02 | [-0.10, -0.01] | -2.64 | 0.008 |
| Portugal | 0.03 | 0.02 | [-0.02, 0.07] | 1.19 | 0.235 |
| Romania | -0.87 | 0.02 | [-0.91, -0.83] | -42.27 | < .001 |
| Sweden | 0.73 | 0.02 | [ 0.68, 0.77] | 32.14 | < .001 |
| Slovenia | 0.01 | 0.02 | [-0.03, 0.05] | 0.57 | 0.569 |
| **Country/Region by time** | . | . | . | . | . |
| Austria by time linear | -0.22 | 0.08 | [-0.38, -0.06] | -2.67 | 0.008 |
| Belgium(VLG) by time linear | -2.37E-03 | 0.07 | [-0.15, 0.14] | -0.03 | 0.975 |
| Belgium(WAL) by time linear | 0.08 | 0.07 | [-0.06, 0.21] | 1.07 | 0.287 |
| Canada by time linear | -0.17 | 0.04 | [-0.25, -0.08] | -3.88 | < .001 |
| Switzerland by time linear | 0.05 | 0.08 | [-0.11, 0.20] | 0.59 | 0.558 |
| Czech Republic by time linear | 0.06 | 0.06 | [-0.07, 0.18] | 0.89 | 0.372 |
| Germany by time linear | -0.19 | 0.08 | [-0.35, -0.02] | -2.22 | 0.026 |
| Denmark by time linear | 0.33 | 0.09 | [ 0.15, 0.52] | 3.57 | < .001 |
| Estonia by time linear | -0.17 | 0.07 | [-0.32, -0.03] | -2.34 | 0.019 |
| Spain by time linear | 0.15 | 0.07 | [ 0.01, 0.29] | 2.06 | 0.04 |
| Finland by time linear | 0.26 | 0.07 | [ 0.12, 0.40] | 3.67 | < .001 |
| France by time linear | 0.34 | 0.06 | [ 0.22, 0.46] | 5.42 | < .001 |
| England by time linear | -0.21 | 0.1 | [-0.40, -0.02] | -2.15 | 0.032 |
| Scotland by time linear | 0.01 | 0.07 | [-0.13, 0.15] | 0.16 | 0.873 |
| Wales by time linear | -0.04 | 0.06 | [-0.15, 0.07] | -0.75 | 0.455 |
| Greenland by time linear | -0.17 | 0.14 | [-0.44, 0.10] | -1.23 | 0.217 |
| Greece by time linear | -0.08 | 0.07 | [-0.22, 0.06] | -1.15 | 0.248 |
| Croatia by time linear | -9.92E-03 | 0.07 | [-0.14, 0.12] | -0.15 | 0.879 |
| Hungary by time linear | -0.17 | 0.07 | [-0.31, -0.02] | -2.3 | 0.022 |
| Ireland by time linear | 0.01 | 0.08 | [-0.14, 0.17] | 0.19 | 0.849 |
| Israel by time linear | 0.57 | 0.09 | [ 0.40, 0.74] | 6.67 | < .001 |
| Iceland by time linear | -0.11 | 0.05 | [-0.22, 0.00] | -2.00 | 0.046 |
| Italy by time linear | 0.18 | 0.08 | [ 0.02, 0.33] | 2.22 | 0.026 |
| Lithuania by time linear | -0.23 | 0.07 | [-0.37, -0.09] | -3.27 | 0.001 |
| Luxembourg by time linear | -0.26 | 0.08 | [-0.42, -0.10] | -3.24 | 0.001 |
| Latvia by time linear | -0.01 | 0.07 | [-0.15, 0.13] | -0.14 | 0.885 |
| North Macedonia by time linear | -0.23 | 0.07 | [-0.38, -0.09] | -3.11 | 0.002 |
| Netherlands by time linear | -0.07 | 0.08 | [-0.23, 0.09] | -0.86 | 0.389 |
| Norway by time linear | 0.07 | 0.09 | [-0.11, 0.25] | 0.80 | 0.421 |
| Poland by time linear | 0.05 | 0.07 | [-0.09, 0.19] | 0.67 | 0.504 |
| Portugal by time linear | -0.18 | 0.07 | [-0.32, -0.03] | -2.41 | 0.016 |
| Romania by time linear | 0.12 | 0.07 | [-0.01, 0.25] | 1.84 | 0.066 |
| Sweden by time linear | 6.52E-03 | 0.08 | [-0.16, 0.17] | 0.08 | 0.939 |
| Slovenia by time linear | 0.22 | 0.07 | [ 0.08, 0.35] | 3.18 | 0.001 |
| **Country/region by time quadratic** | . | . | . | . | . |
| Austria by time quadratic | 0.09 | 0.06 | [-0.03, 0.20] | 1.48 | 0.14 |
| Belgium(VLG) by time quadratic | 0.01 | 0.05 | [-0.08, 0.11] | 0.24 | 0.807 |
| Belgium(WAL) by time quadratic | 0.16 | 0.05 | [ 0.07, 0.25] | 3.42 | < .001 |
| Canada by time quadratic | 0.01 | 0.04 | [-0.06, 0.08] | 0.35 | 0.73 |
| Switzerland by time quadratic | -0.13 | 0.06 | [-0.24, -0.01] | -2.19 | 0.028 |
| Czech Republic by time quadratic | 0.25 | 0.05 | [ 0.16, 0.35] | 5.44 | < .001 |
| Germany by time quadratic | -0.10 | 0.05 | [-0.21, 0.00] | -2.02 | 0.043 |
| Denmark by time quadratic | 0.13 | 0.06 | [ 0.01, 0.24] | 2.13 | 0.033 |
| Estonia by time quadratic | 0.16 | 0.05 | [ 0.06, 0.26] | 3.05 | 0.002 |
| Spain by time quadratic | -0.04 | 0.04 | [-0.12, 0.05] | -0.83 | 0.409 |
| Finland by time quadratic | -0.22 | 0.05 | [-0.32, -0.13] | -4.47 | < .001 |
| France by time quadratic | -0.29 | 0.05 | [-0.39, -0.20] | -6.14 | < .001 |
| England by time quadratic | -0.06 | 0.06 | [-0.17, 0.05] | -1.04 | 0.299 |
| Scotland by time quadratic | -0.27 | 0.05 | [-0.37, -0.17] | -5.2 | < .001 |
| Wales by time quadratic | -0.08 | 0.04 | [-0.16, 0.01] | -1.79 | 0.074 |
| Greenland by time quadratic | -0.31 | 0.1 | [-0.50, -0.11] | -3.11 | 0.002 |
| Greece by time quadratic | 0.10 | 0.05 | [ 0.00, 0.20] | 2.02 | 0.043 |
| Croatia by time quadratic | 0.04 | 0.05 | [-0.05, 0.14] | 0.91 | 0.363 |
| Hungary by time quadratic | 0.19 | 0.05 | [ 0.09, 0.30] | 3.53 | < .001 |
| Ireland by time quadratic | -0.07 | 0.05 | [-0.18, 0.04] | -1.3 | 0.193 |
| Israel by time quadratic | -0.01 | 0.06 | [-0.12, 0.10] | -0.18 | 0.855 |
| Iceland by time quadratic | -0.09 | 0.04 | [-0.16, -0.01] | -2.35 | 0.019 |
| Italy by time quadratic | 0.09 | 0.06 | [-0.02, 0.21] | 1.63 | 0.103 |
| Lithuania by time quadratic | 0.02 | 0.05 | [-0.07, 0.11] | 0.45 | 0.655 |
| Luxembourg by time quadratic | -0.06 | 0.06 | [-0.17, 0.06] | -0.96 | 0.338 |
| Latvia by time quadratic | 0.26 | 0.05 | [ 0.17, 0.36] | 5.66 | < .001 |
| North Macedonia by time quadratic | 0.02 | 0.05 | [-0.08, 0.13] | 0.48 | 0.628 |
| Netherlands by time quadratic | 0.04 | 0.06 | [-0.08, 0.16] | 0.66 | 0.507 |
| Norway by time quadratic | 5.38E-03 | 0.06 | [-0.12, 0.13] | 0.09 | 0.932 |
| Poland by time quadratic | -0.13 | 0.05 | [-0.23, -0.04] | -2.73 | 0.006 |
| Portugal by time quadratic | -0.03 | 0.05 | [-0.13, 0.07] | -0.59 | 0.554 |
| Romania by time quadratic | 0.26 | 0.05 | [ 0.17, 0.35] | 5.54 | < .001 |
| Sweden by time quadratic | 0.23 | 0.06 | [ 0.11, 0.35] | 3.83 | < .001 |
| Slovenia by time quadratic | -0.08 | 0.05 | [-0.17, 0.02] | -1.6 | 0.109 |
| **Country/region by time cubic** | . | . | . | . | . |
| Austria by time cubic | 0.20 | 0.14 | [-0.07, 0.47] | 1.44 | 0.15 |
| Belgium(VLG) by time cubic | 0.39 | 0.13 | [ 0.14, 0.63] | 3.07 | 0.002 |
| Belgium(WAL) by time cubic | -0.21 | 0.12 | [-0.45, 0.03] | -1.71 | 0.088 |
| Canada by time cubic | -0.18 | 0.08 | [-0.34, -0.03] | -2.29 | 0.022 |
| Switzerland by time cubic | -0.44 | 0.14 | [-0.71, -0.17] | -3.18 | 0.001 |
| Czech Republic by time cubic | -0.15 | 0.11 | [-0.36, 0.06] | -1.37 | 0.172 |
| Germany by time cubic | 0.05 | 0.14 | [-0.23, 0.33] | 0.33 | 0.74 |
| Denmark by time cubic | -0.64 | 0.16 | [-0.95, -0.33] | -4.09 | < .001 |
| Estonia by time cubic | 0.17 | 0.13 | [-0.08, 0.42] | 1.33 | 0.184 |
| Spain by time cubic | -0.17 | 0.12 | [-0.42, 0.07] | -1.41 | 0.16 |
| Finland by time cubic | 0.05 | 0.12 | [-0.19, 0.29] | 0.43 | 0.668 |
| France by time cubic | -0.39 | 0.11 | [-0.60, -0.17] | -3.52 | < .001 |
| England by time cubic | 0.27 | 0.16 | [-0.05, 0.59] | 1.68 | 0.093 |
| Scotland by time cubic | -0.19 | 0.13 | [-0.43, 0.06] | -1.5 | 0.134 |
| Wales by time cubic | -0.1 | 0.1 | [-0.29, 0.09] | -1.01 | 0.311 |
| Greenland by time cubic | 0.58 | 0.24 | [ 0.11, 1.05] | 2.42 | 0.015 |
| Greece by time cubic | 0.46 | 0.12 | [ 0.22, 0.71] | 3.78 | < .001 |
| Croatia by time cubic | 0.22 | 0.11 | [ 0.00, 0.44] | 1.92 | 0.055 |
| Hungary by time cubic | 0.18 | 0.13 | [-0.08, 0.43] | 1.36 | 0.173 |
| Ireland by time cubic | 0.21 | 0.13 | [-0.05, 0.47] | 1.59 | 0.112 |
| Israel by time cubic | -0.53 | 0.14 | [-0.81, -0.24] | -3.66 | < .001 |
| Iceland by time cubic | 0.28 | 0.09 | [ 0.09, 0.46] | 2.96 | 0.003 |
| Italy by time cubic | -0.46 | 0.14 | [-0.73, -0.19] | -3.3 | < .001 |
| Lithuania by time cubic | 0.71 | 0.12 | [ 0.48, 0.95] | 5.93 | < .001 |
| Luxembourg by time cubic | 0.33 | 0.14 | [ 0.06, 0.60] | 2.38 | 0.018 |
| Latvia by time cubic | -0.08 | 0.12 | [-0.32, 0.16] | -0.68 | 0.495 |
| Macedonia by time cubic | 0.36 | 0.13 | [ 0.11, 0.61] | 2.79 | 0.005 |
| Netherlands by time cubic | -0.14 | 0.14 | [-0.43, 0.14] | -1.00 | 0.319 |
| Norway by time cubic | -0.49 | 0.16 | [-0.79, -0.18] | -3.13 | 0.002 |
| Poland by time cubic | -0.41 | 0.12 | [-0.65, -0.17] | -3.29 | < .001 |
| Portugal by time cubic | 0.51 | 0.13 | [ 0.26, 0.75] | 3.99 | < .001 |
| Romania by time cubic | -0.20 | 0.11 | [-0.42, 0.03] | -1.73 | 0.085 |
| Sweden by time cubic | -0.08 | 0.15 | [-0.38, 0.21] | -0.55 | 0.584 |
| Slovenia by time cubic | -0.03 | 0.12 | [-0.26, 0.20] | -0.24 | 0.813 |

Note. The regression coefficients for Slovakia, were redundant in the regression model, due to the deviation contrast specification. The estimate for Slovakia can be derived as the negative of the sum of deviations for the other countries.

Supplementary table S4.

Model coefficients for random effects approach, model 4b. Time is centred on year 2016 and scaled to 10-year unit. Regression coefficients for time are on a logit scale, reflecting change per 10-year period. (Health Behaviour in School-aged Children study, 2006-2022).

| **Variable** | **Coef.** | **SE** | **t** | **p-value** |
| --- | --- | --- | --- | --- |
| Fixed |  |  |  |  |
| Intercept) | 0.795 | 0.033 | 23.80 | <0.001 |
| Time | 0.134 | 0.018 | 7.32 | <0.001 |
| Time-quadratic | -0.125 | 0.020 | -6.11 | <0.001 |
| Random |  |  |  |  |
| Country/Region-year SD intercept(U0) | 0.079 |  |  |  |
| Country/Region SD intercept (V0) | 0.421 |  |  |  |
| cor(V1time, V0 intercept) | -0.552 |  |  |  |
| Country/Region SD (V1time) | 0.123 |  |  |  |

Note. N student=955694, n region-year=175, n region=35


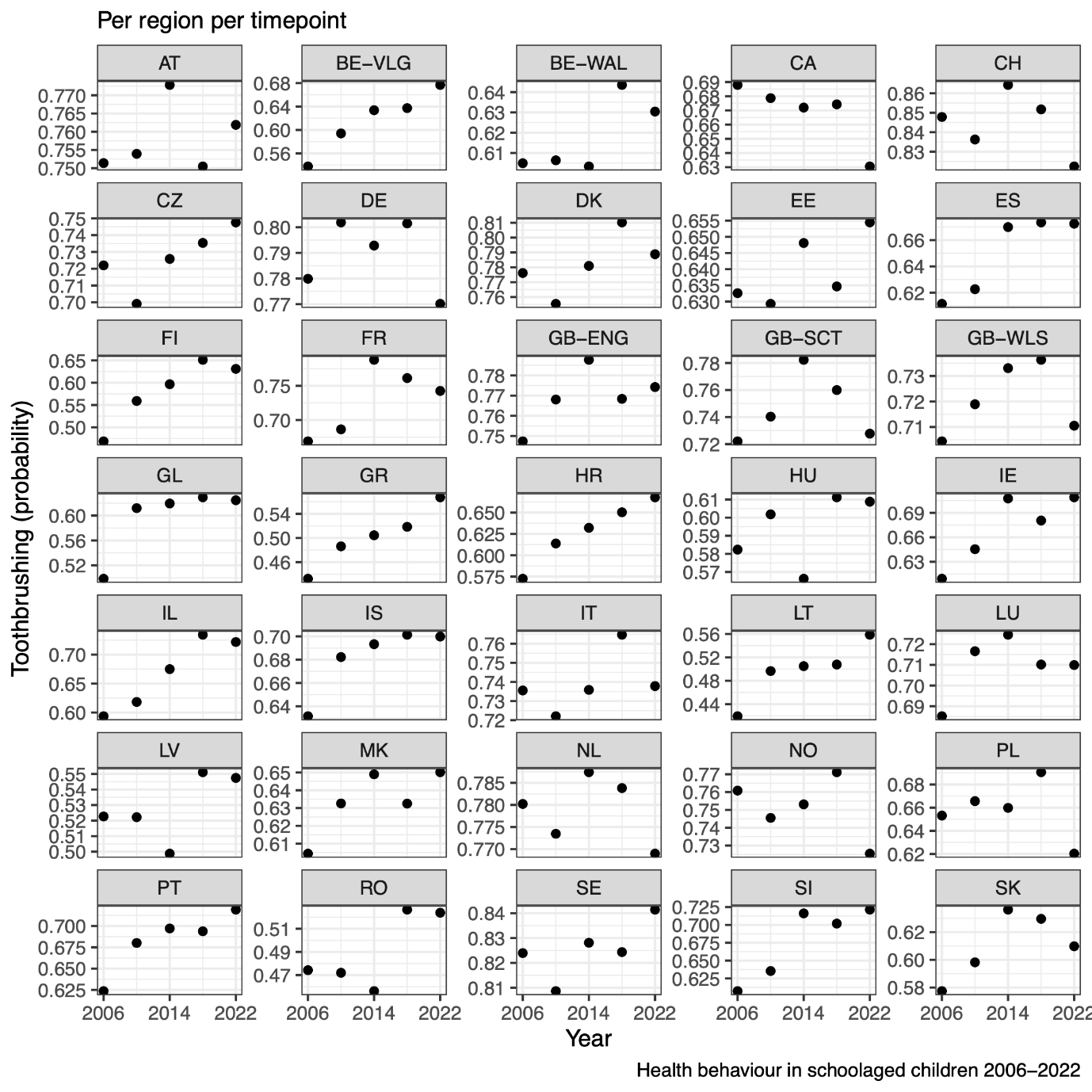


*Supplementary Figure S1.*

*Faceted scatterplot of region prevalence of toothbrushing by time 2006–2022. Each dot represents one country or regions prevalence in a given year, y-scale different for all regions (Data from Health Behaviour in School-aged Children study, 2006–2022).*

*
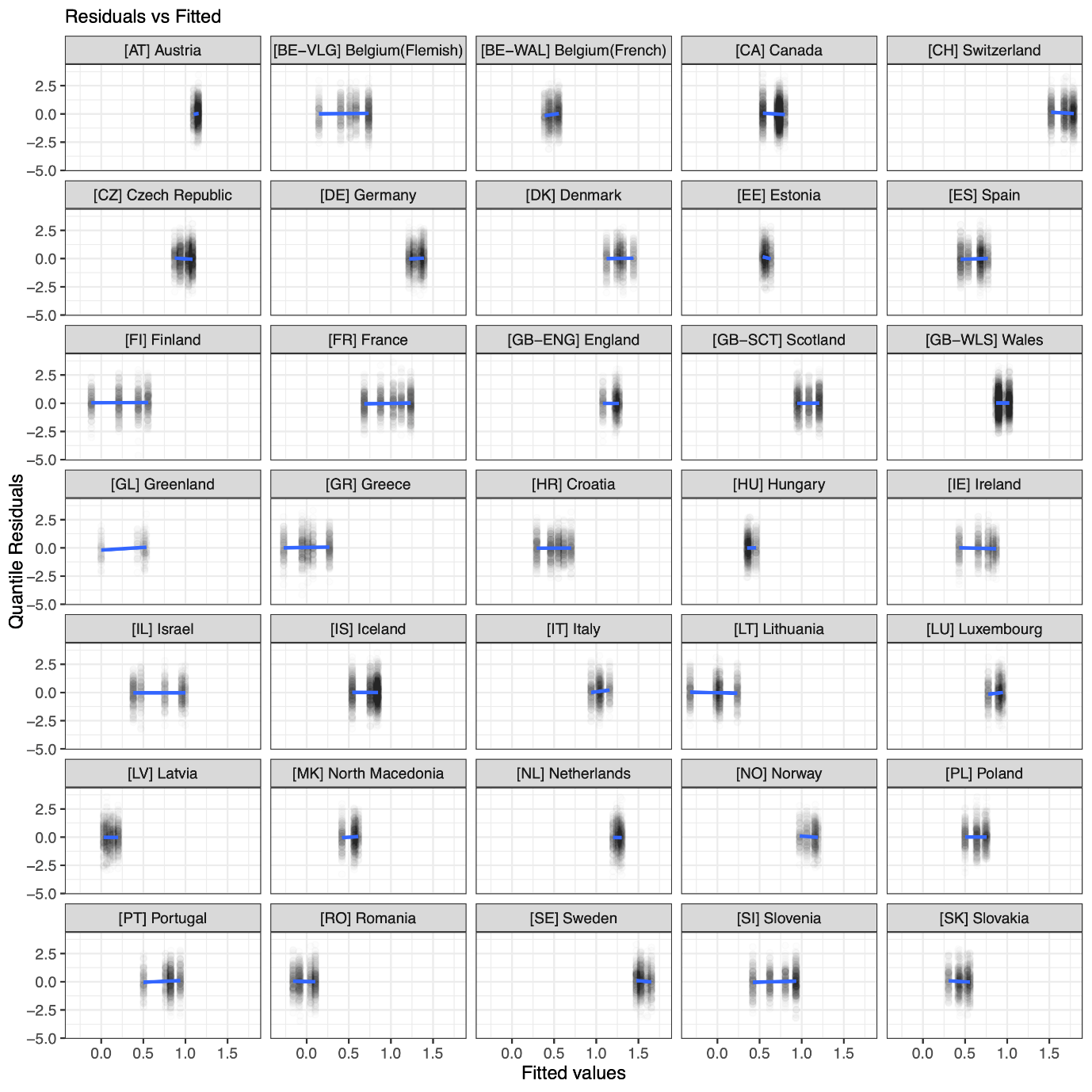
*

*Supplementary Figure S2 | Faceted model diagnostic plot for stratified approach*

*model M3 - cubic effect, with quantile residuals of on y-axis and linear prediction of*

*toothbrushing per country or region on x-axis (Data from Health Behaviour in School-aged Children study, 2006–2022).*

*
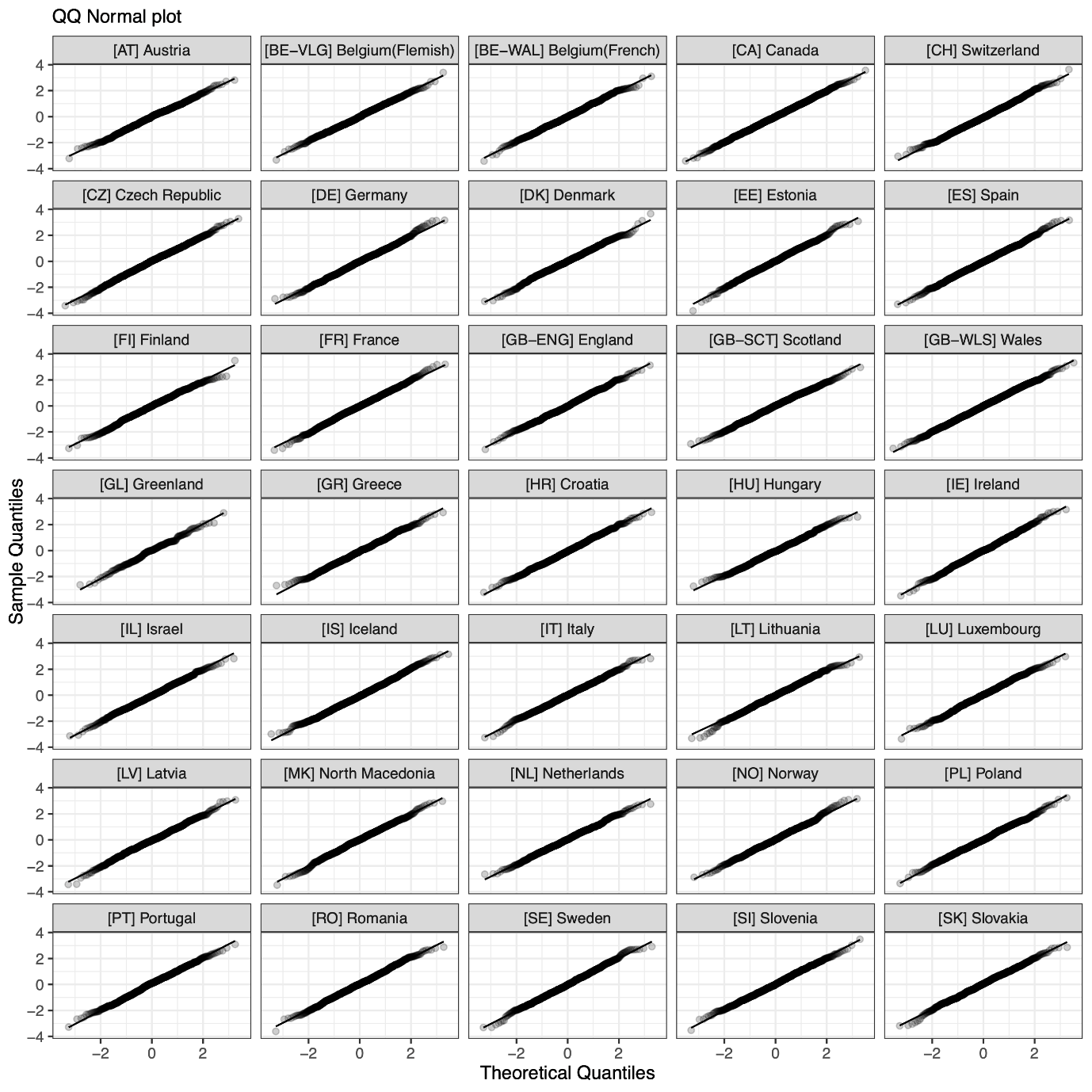
*

*Supplementary Figure S3*

*Faceted Quantile-Quantile plot of quantile residuals for stratified approach Model M3 cubic effects of time against normal theory distribution per country or region (Data from Health Behaviour in School-aged Children study, 2006–2022).*

*
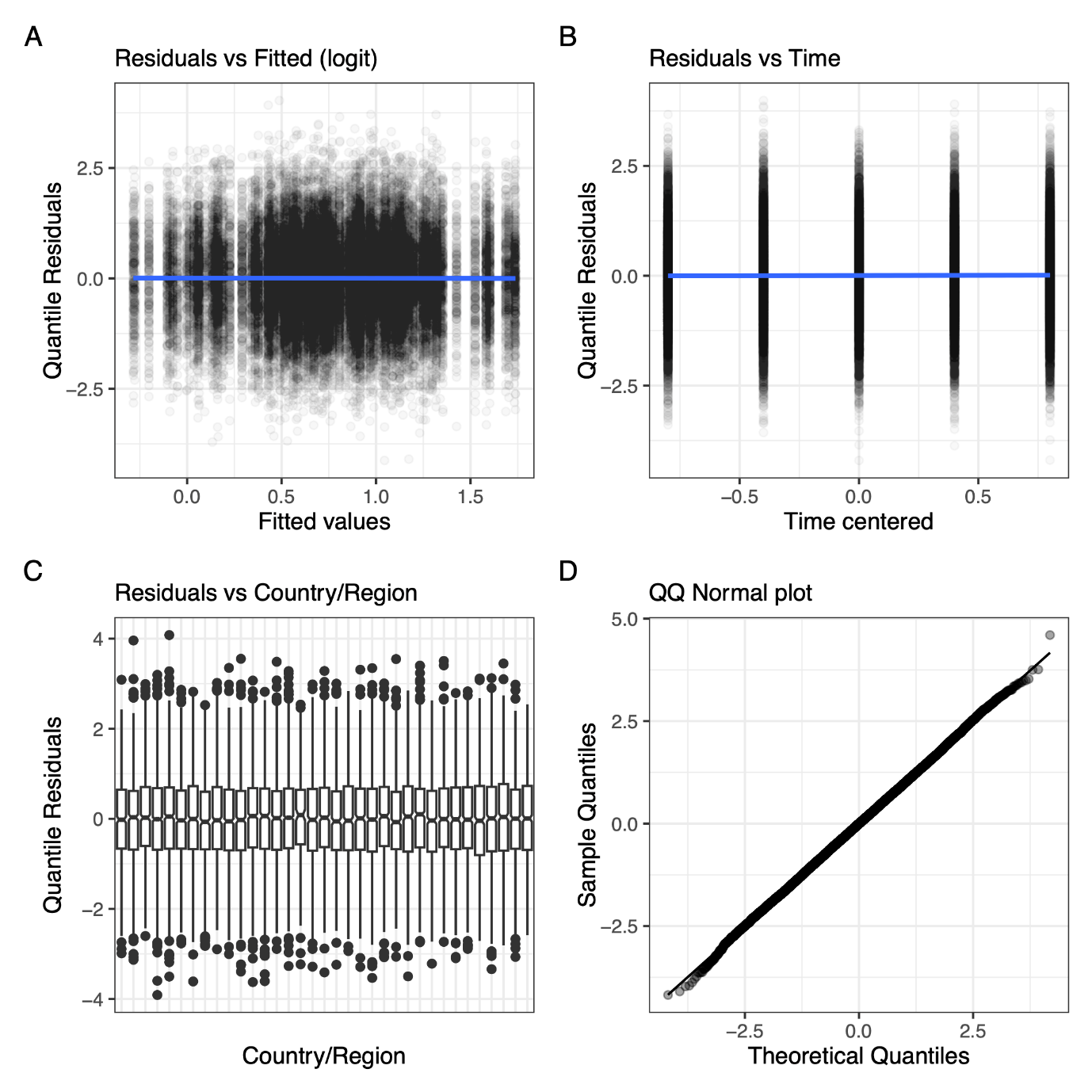
*

*Supplementary Figure S4*

*Model diagnostic panel for fixed effects approach model M6: (A) Quantile residuals against linear predictor, (B) quantile residual and time, (C) quantile residuals and country/region, (D) Normal quantile-quantile plot of quantile residuals and theoretical expectation (Data from Health Behaviour in School-aged Children study, 2006–2022).*

*
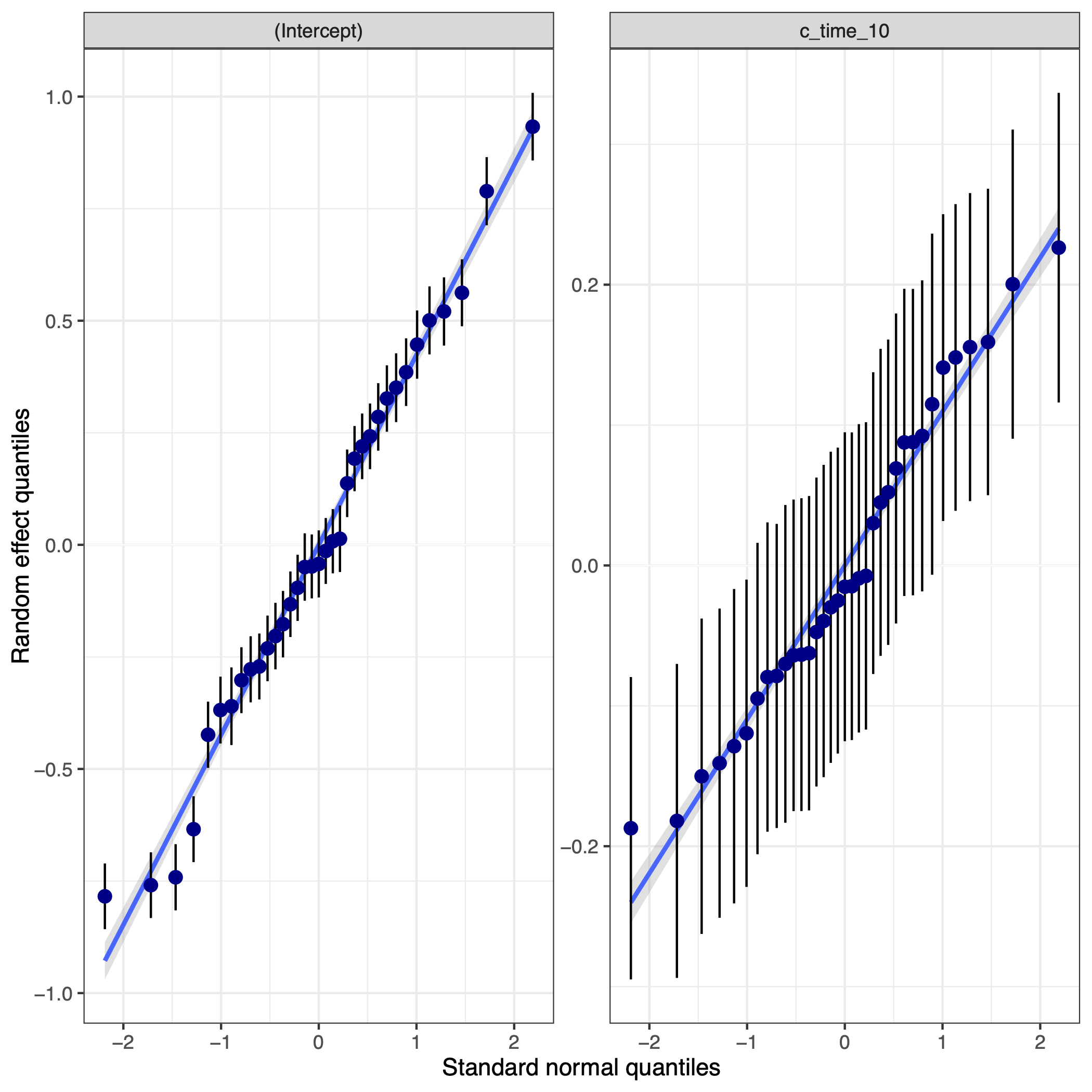
*

*Supplementary Figure S5*

*Quantile-quantile plot of random effects Country/region level, with 95% CI for the effect (Data from Health Behaviour in School-aged Children study, 2006–2022).*

*
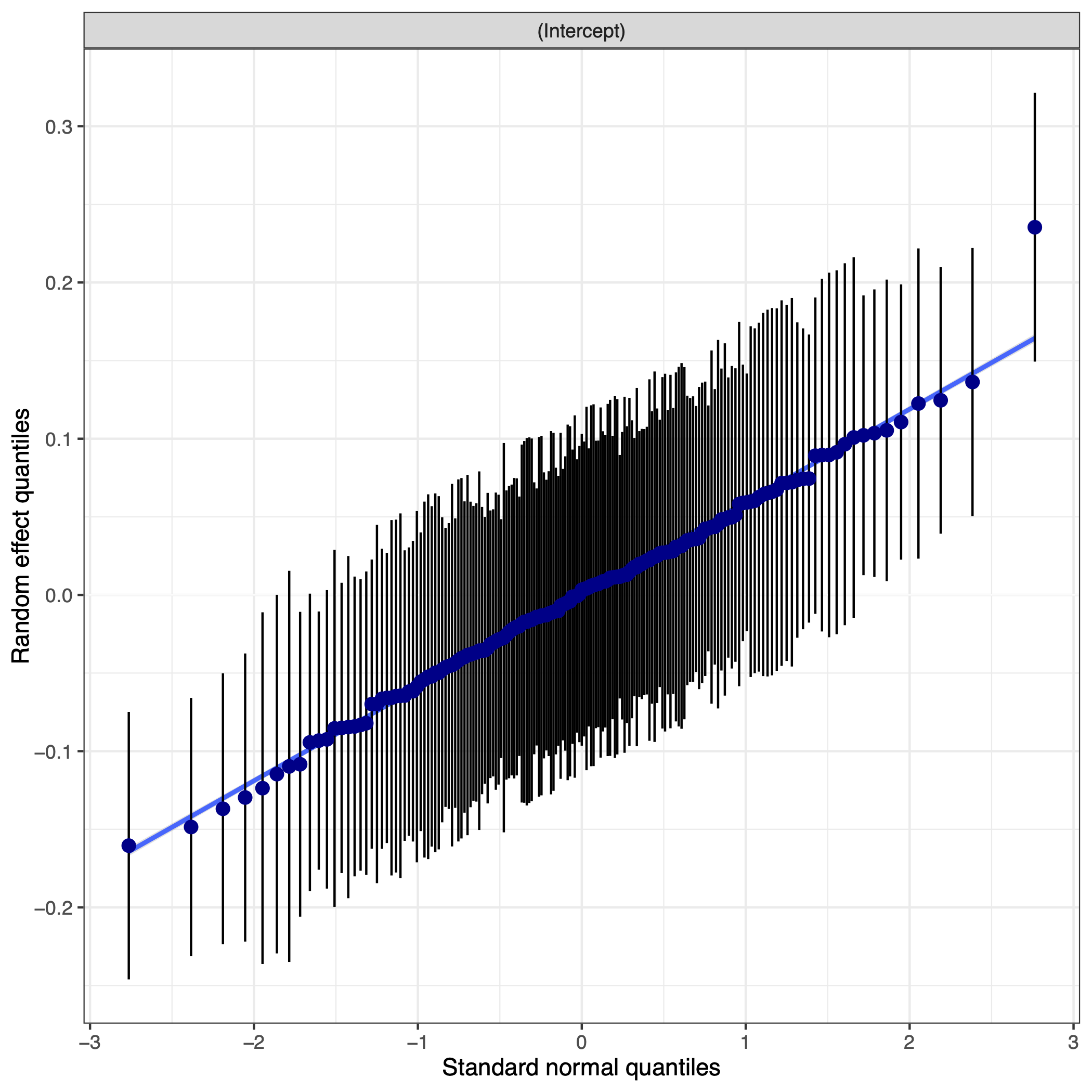
*

*Supplementary Figure S6*

*Quantile-quantile plot of random effects at Country/region-year level, with 95% CI for the effect (Data from Health Behaviour in School-aged Children study, 2006–2022).*
